# The Transcriptional Stress Response of *Candida albicans* to Weak Organic Acids

**DOI:** 10.1534/g3.114.015941

**Published:** 2015-01-29

**Authors:** Fabien Cottier, Alrina Shin Min Tan, Jinmiao Chen, Josephine Lum, Francesca Zolezzi, Michael Poidinger, Norman Pavelka

**Affiliations:** * Singapore Immunology Network (SIgN), Agency for Science, Technology and Research (A*STAR), Singapore 138648, Singapore; † Department of Biological Sciences, National University of Singapore, Singapore 117543, Singapore; ‡ School of Biological Sciences, Nanyang Technological University, Singapore 637551, Singapore

**Keywords:** short-chain fatty acid, transcriptomics, gene expression profiling, RNA-seq, power law global error model

## Abstract

*Candida albicans* is the most important fungal pathogen of humans, causing severe infections, especially in nosocomial and immunocompromised settings. However, it is also the most prevalent fungus of the normal human microbiome, where it shares its habitat with hundreds of trillions of other microbial cells. Despite weak organic acids (WOAs) being among the most abundant metabolites produced by bacterial microbiota, little is known about their effect on *C. albicans*. Here we used a sequencing-based profiling strategy to systematically investigate the transcriptional stress response of *C. albicans* to lactic, acetic, propionic, and butyric acid at several time points after treatment. Our data reveal a complex transcriptional response, with individual WOAs triggering unique gene expression profiles and with important differences between acute and chronic exposure. Despite these dissimilarities, we found significant overlaps between the gene expression changes induced by each WOA, which led us to uncover a core transcriptional response that was largely unrelated to other previously published *C. albicans* transcriptional stress responses. Genes commonly up-regulated by WOAs were enriched in several iron transporters, which was associated with an overall decrease in intracellular iron concentrations. Moreover, chronic exposure to any WOA lead to down-regulation of RNA synthesis and ribosome biogenesis genes, which resulted in significant reduction of total RNA levels and of ribosomal RNA in particular. In conclusion, this study suggests that gastrointestinal microbiota might directly influence *C. albicans* physiology via production of WOAs, with possible implications of how this fungus interacts with its host in both health and disease.

*Candida albicans* is the fungal pathogen most frequently associated with human mycoses, ranging from superficial lesions of the skin and mucosae to life-threatening systemic infections, especially in immunocompromised individuals (Pfaller and Diekema 2007). Despite its negative reputation, this organism is also the most widely associated fungus across the general population, colonizing all body surfaces, including the vaginal mucosa and the gastrointestinal tract (GI) of most healthy individuals (MacCallum 2010), suggesting it to be a member of the commensal microbiota. Of note, bloodstream infections by *C. albicans* often originate from the same strain carried in the GI tract of the respective patients (Miranda *et al.* 2009), indicating an opportunistic nature of this pathogen. However, what precisely controls the switch between asymptomatic colonization and invasive fungal infection is not understood fully.

Bacterial−fungal interactions occur in all niches of the human body, and some of them have been described to influence pathogenesis and disease outcomes (Peleg *et al.* 2010). For instance, probiotic lactobacilli were reported to induce immunomodulatory effects protecting mice from subsequent *C. albicans* infection (Villena *et al.* 2011; Zelante *et al.* 2013). But *C. albicans* also directly responds to bacteria-derived metabolites like quorum-sensing molecules or muramyl dipeptides, which have been described to respectively inhibit or promote the yeast-to-hyphal transition, an important virulence factor of *C. albicans* (Lo *et al.* 1997; Hall *et al.* 2011; Xu *et al.* 2008). Among the most abundant metabolites of bacterial origin that can be found on mucosal surfaces of the human body are weak organic acids (WOAs), such as lactic, acetic, propionic, and butyric acid. However, little is known about the effect of these molecules on *C. albicans*.

On the vaginal mucosa, the large number of lactic acid-producing bacteria, especially members of the *Lactobacillus* genus (Cribby *et al.* 2008), correlates with a significant concentration of lactic acid (~55−111 mM) (O’Hanlon *et al.* 2011), which is thought to contribute to the control of *C. albicans* colonization in healthy women (Osset *et al.* 2001; Kohler *et al.* 2012). The human GI tract harbors the largest number and variety of bacterial species of the entire human microbiome (Arumugam *et al.* 2011; Qin *et al.* 2010). Among the most abundant metabolites that can be found in the gut are the short-chain fatty acids (SCFAs) acetic, propionic and butyric acid, which are primarily the result of bacterial anaerobic fermentation (Mortensen and Clausen 1996) and that can reach total concentrations of up to 140 mM in the proximal colon (Topping and Clifton 2001). Whereas acetic acid is produced by a large spectrum of bacteria, clostridial clusters IV and XIV are known to specifically produce butyric acid, and the *Propionibacterium* genus has been described to synthesize propionic acid (Cousin *et al.* 2012). It was reported that a single concentration of acetic, propionic or butyric acid reduced *C. albicans* colony formation (Huang *et al.* 2011), but the mechanism of action was not addressed. In addition, butyric acid was reported to inhibit *C. albicans* yeast-to-hyphal transition, albeit only at relatively high concentrations, while acetic and propionic acid had no such effect (Noverr and Huffnagle 2004).

In the present study, we aimed to investigate the effect of lactic, acetic, propionic, and butyric acid on the *C. albicans* transcriptome. By means of RNA-sequencing experiments, we found unique gene expression profiles for each WOA and large differences between acute and chronic exposure. Nevertheless, we identified a core transcriptional stress response common to all tested acids that correctly predicted decreased intracellular iron concentrations and decreased RNA synthesis and ribosome biogenesis. These results suggest that WOA-producing bacteria of the human microbiome might directly modulate important physiological parameters of *C. albicans*.

## Materials and Methods

### Strains and culture conditions

The wild-type *Candida albicans* strain SC5314 (Noble and Johnson 2005) was used throughout this study. Stock cultures were preserved in 35% glycerol and maintained at −80°. Cells were grown in De Man, Rogosa, and Sharpe (MRS) media (Sigma-Aldrich) at 37° in a shaking incubator at 150−200 rpm. MRS medium was chosen, because it was previously shown to support the growth of both *C. albicans* (Kohler *et al.* 2012) and lactobacilli (De Man *et al.* 1960). Individual acids, *i.e.*, hydrochloric, lactic, acetic, propionic, or butyric acid (from either Merck or Sigma-Aldrich), were added to freshly relaunched overnight cultures diluted down to an optical density (OD) at 600 nm of 0.1. The prototroph *sfu1*Δ mutant (SN668) and its isogenic control (SN425) were kindly provided by Suzanne Noble (Noble *et al.* 2010).

### RNA sequencing

Cells were harvested by centrifugation, and the pellet was flash-frozen in liquid nitrogen. RNA extractions were performed following a previously described protocol (Pavelka *et al.* 2010). Total RNA yield was quantified with the Quant-iT RiboGreen RNA Assay Kit (Invitrogen) and RNA quality assessed on the 2100 Bioanalyzer (Agilent). Relative abundance of ribosomal RNA was estimated based on the area under the electropherogram peaks predicted to correspond to the 18S and 28S rRNA molecules. All the 96 total RNA samples had an RNA integrity number ≥7, of which 81% had RNA integrity number ≥8. Complementary DNA (cDNA) libraries were prepared with 300 ng of total RNA and 2 µL of a 3:1000 dilution of ERCC RNA Spike in Controls (Ambion). The fragmented mRNA samples were subjected to cDNA synthesis using Illumina TruSeq RNA sample preparation kit version 2 (Low-Throughput protocol) according to manufacturer’s protocol, except for the following modifications: (1) use of 12 polymerase chain reaction cycles; (2) two additional rounds of Agencourt Ampure XP SPRI beads (Beckman Courter) to remove >600 bp double-stranded cDNA. The length distribution of the cDNA libraries was monitored with DNA 1000 kits on the Agilent Bioanalyzer. All samples were subjected to an indexed paired-end sequencing run of 2 × 51 cycles on an Illumina HiSeq 2000 (12 libraries/lane). Raw and processed sequencing data from this article have been deposited with the Gene Expression Omnibus database (http://www.ncbi.nlm.nih.gov/geo/) under accession no. GSE49310.

### Data analysis

Raw sequencing reads were first mapped to the ERCC spike-in control sequences with bowtie2 (Langmead *et al.* 2009). For each run, the mate distance and standard deviation were determined with Picard (http://picard.sourceforge.net), and these values used to map the reads to the reference genome using TopHat (Trapnell *et al.* 2009), which was run using the *Candida albicans* genome annotation (Assembly 21, version s02-m05-r10) as a guide. The number of reads mapping to each transcript in the genome was counted with an in-house R script. Reads mapping to overlapping regions between multiple transcripts were assigned to unique transcripts based on information from the uniquely mapping portion of each transcript and by assuming an additive effect of the read depth in the overlapping regions. Assigned read counts were then converted into reads per kilobase per million mapped reads (RPKMs). Principal component analysis and complete-linkage hierarchical clustering were performed using built-in R functions (R Core Team 2013) on log_2_-transformed RPKM values followed by z-score normalization of the gene expression profiles. Differentially expressed transcripts were identified with R package ‘plgem’ (Pavelka *et al.* 2004), which we used to compare acid-treated samples to time-matched untreated samples. For PLGEM analysis, raw RPKM values were normalized by dividing each value by the mean RPKM value of the corresponding sample. Resampled statistics were obtained via 10,000 random iterations, and a significance threshold of 0.0005 (predicted to select ~3 false-positive hits per sample) was chosen to identify differentially expressed genes. Gene Ontology (GO) enrichment analysis was performed with the CGD Gene Ontology Term Finder (Inglis *et al.* 2012), with *P* values corresponding to Bonferroni-corrected hypergeometric test *P* values. The magnitude of the overlap between two gene lists was evaluated as the ratio of the intersection over the union of the two lists. Statistical significance of the gene lists’ overlap was evaluated by a series of Fisher exact tests. All other statistical analyses were performed in Microsoft Excel 2007 or Prism (GraphPad) and, unless otherwise indicated in the respective figure legends, we used unpaired *t*-tests assuming unequal variances and error bars corresponded to standard errors of the mean.

### Determination of iron concentration

A bathophenanthrolinedisulfonic acid-based colorimetric method was used to determine both the extracellular and the intracellular iron concentrations of *C. albicans* as described previously (Hsu *et al.* 2011). Absorbance measurements at 535, 600, and 680 nm were performed on a SmartSpec Plus spectrophotometer (Bio-Rad).

## Results

### Global effect of WOAs on the *C. albicans* transcriptome

**Figure 1:**
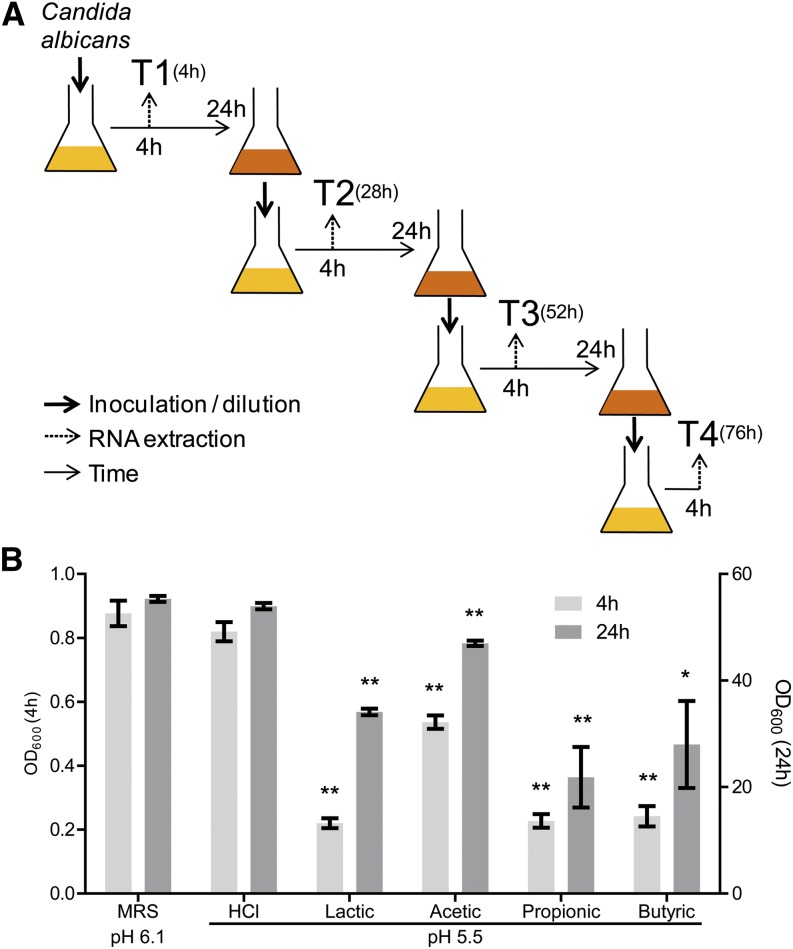
Acute and chronic exposure of *Candida albicans* to lactic, acetic, propionic, and butyric acid: (A) Overall scheme of the transcriptional profiling experiment. *C. albicans* overnight cultures were diluted in fresh media every 24 hr and samples for RNA extraction were collected 4 hr after each dilution. (B) OD_600nm_ of the culture was measured at 4 hr and 24 hr after *C. albicans* inoculation in media supplemented with the indicated acids (all adjusted to pH 5.5 with the addition of NaOH) and compared with untreated control cultures (De Man, Rogosa, and Sharpe medium [MRS] at pH 6.1). n = 5; *P < 0.05 **P < 0.01. OD, optical density.

To shed light on the overall cellular response of *C. albicans* to WOAs, we used a genome-wide transcriptional profiling approach based on cDNA deep sequencing. Because *C. albicans* is expected to encounter these bacterial-derived metabolites in the GI tract over prolonged periods of time, we performed the analysis not only on acute but also on several time points of chronic exposure of each individual acid for up to ~3 d (Figure 1A). Four independent *C. albicans* cultures were grown under each of the following six conditions (for a total of 24 independent cultures): MRS at pH 6.1 (unmodified medium), MRS at pH 5.5 (medium acidified by addition of HCl), or MRS supplemented with 622 mM lactic, 75 mM acetic, 52 mM propionic, or 18 mM butyric acid, all adjusted to pH 5.5 by the addition of NaOH. These WOA concentrations were empirically chosen for their ability to significantly affect the OD of the cultures at both early (4 hr) and late (24 hr) time points compared with untreated or HCl-treated cultures (Figure 1B). To ensure that each culture was analyzed in the early exponential phase of growth, we used a daily serial passaging design, in which samples were harvested on each day at the same time (*i.e.*, 4 hr) after we relaunched the previous overnight culture into fresh medium. Four time points (termed T1−T4), corresponding to each of four subsequent serial passages, were chosen for transcriptome analysis.

*C. albicans* cDNA libraries were prepared starting from total RNA for a total of 96 independent samples (four time points for each of 24 independent cultures) and sequenced on an Illumina HiSequation 2000 platform. RNA spike-ins displayed the expected fold-changes (data not shown). We obtained high-quality, high-coverage transcriptome information with ~22 million sequencing reads per sample uniquely mapped to 6453 transcripts, including both open-reading frames and noncoding RNAs. We validated that no chromosome-specific coverage biases were present in any of the samples (Supporting Information, Figure S1), therefore excluding the possibility that interesting gene expression patterns might be a consequence of aneuploidy accumulated prior to or during the course of the experiment. A total of seven samples were detected as outliers by a combination of principal component analysis and hierarchical clustering for being significantly different from their corresponding biological replicates (data not shown) and were therefore discarded from all subsequent analyses.

**Figure 2:**
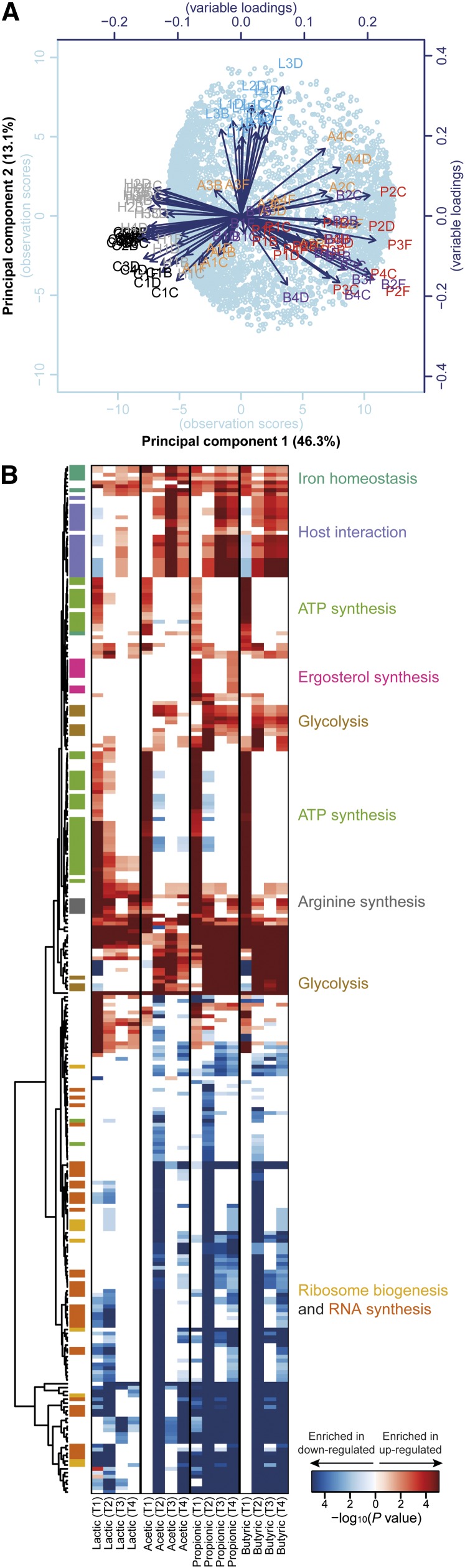
Global transcriptome changes induced by weak organic acids (WOAs) in *Candida albicans*: (A) Principal component analysis of the entire transcriptome dataset of 6453 transcripts across 89 conditions. Samples are plotted by their first two principal components. The first letter of the sample name represents the treatment (C: De Man, Rogosa, and Sharpe medium [MRS] control, H: HCl, L: lactic, A: acetic, P: propionic, B: butyric acid), the middle number represents the time point (1: T1, 2: T2, 3: T3, 4: T4), whereas the last letter refers to the biological replicate (B, C, D, or F). (B) Hierarchical clustering of Gene Ontology (GO) Biological Process terms based on their enrichment in lists of genes differentially expressed in response to individual WOAs and individual time points. Before enrichment analysis, differentially expressed gene lists were removed of genes also significantly modulated in HCl-treated *vs.* untreated cells. The heat map is color-coded based on Bonferroni-corrected *P* values, as indicated by the color bar at the bottom. Only GO terms with an adjusted *P* value < 5 × 10^−4^ in at least one of the 16 conditions were included in the heat map. Related GO terms are color-coded based on the more general functional category indicated on the right. Distance metric: Euclidean.

Unsupervised principal component analysis (Figure 2A) and hierarchical clustering (Figure S2) of the 89 analyzed samples led to the following observations. Consistent with the effect on the OD of the cultures (Figure 1B), the HCl-treated samples clustered more closely with the untreated samples than with any other samples, indicating that the effect exerted by WOAs on *C. albicans* goes beyond a mere response to acidification of the media. Lactic acid−treated samples formed a cluster by themselves that was relatively compact through time, suggesting a unique cellular response to this acid. Acetic acid−treated cells were closer to control cells at T1 but clustered away from all other samples at T3 and T4, indicating a distinct feature in the transcriptional response of *C. albicans* to this particular acid especially under chronic exposure. Also, propionic and butyric acid displayed a differential behavior between T1 and subsequent time points; however, they also were consistently more similar to each other than to any other treatment.

### Common and specific gene expression changes in *C. albicans* treated with WOAs

To further understand the transcriptional response of *C. albicans* to WOAs, we next performed a supervised analysis to focus on genes significantly regulated in presence of each of the four WOAs at each of the four time points but that were not significantly regulated by HCl. To this end, we first fitted each set of biological replicates to a power law global error model (PLGEM), previously shown to faithfully describe the variance-*vs.*-mean dependence that exists in replicated high-density oligonucleotide microarray datasets as well as mass spectrometry-based proteomics datasets (Pavelka *et al.* 2004, 2008). Here we report for the first time that a PLGEM can be used to model also replicated RNA sequencing-based transcriptomic datasets with excellent goodness-of-fit (Figure S3). Interestingly, the PLGEM slopes obtained in this RNA-sequencing dataset were all between 0.5 and 1, *i.e.*, in the same range of previously described types of data. Hence, we proceeded with the PLGEM analysis pipeline to detect differentially expressed genes at each time point in comparison to time-matched untreated controls and removed genes found to be significantly changing in response to HCl treatment to eliminate transcriptional responses due only to low pH (File S1).

We next proceeded with a global functional characterization of these lists of differentially expressed genes, to gain biological insights into the transcriptional responses of *C. albicans* to different WOAs at different time points. To this end, we first obtained statistically significantly enriched GO Biological Process terms among the transcripts significantly up- or down-regulated at each time point and in response to each WOA (File S2). We then clustered these GO terms based on the enrichment scores obtained across each condition (Figure 2B). This analysis revealed several biological processes that were commonly regulated across several different WOAs, as well as processes more specifically modulated by only some WOA or only at a few time points. In general, a richer spectrum of biological processes was identified among the up-regulated genes compared with the down-regulated genes. Among the processes commonly up-regulated by all tested WOAs, iron homeostasis was enriched at all time-points, whereas ATP and arginine biosynthesis mostly at earlier time points. Genes involved in glycolysis and in interaction with the host were commonly up-regulated by acetic, propionic, and butyric acid, most especially at later time points. Ergosterol biosynthesis, on the other hand, was specifically enriched only in genes up-regulated by propionic acid and, curiously, only at T1 and T4. Conversely, within the set of down-regulated genes most biological processes were linked to either RNA synthesis or ribosome biogenesis. Although many of these GO terms were only found to be enriched in response to acetic, propionic, and butyric acid, several RNA synthesis and ribosome biogenesis biological processes were also found in response to lactic acid, albeit often at different time points for different acids. Overall, this analysis revealed a complex portrait of the transcriptional response of *C. albicans* to WOAs, with a few important functional differences between acids and between time points, but also several commonly regulated biological processes.

### The core transcriptional response of *C. albicans* to WOAs

**Figure 3:**
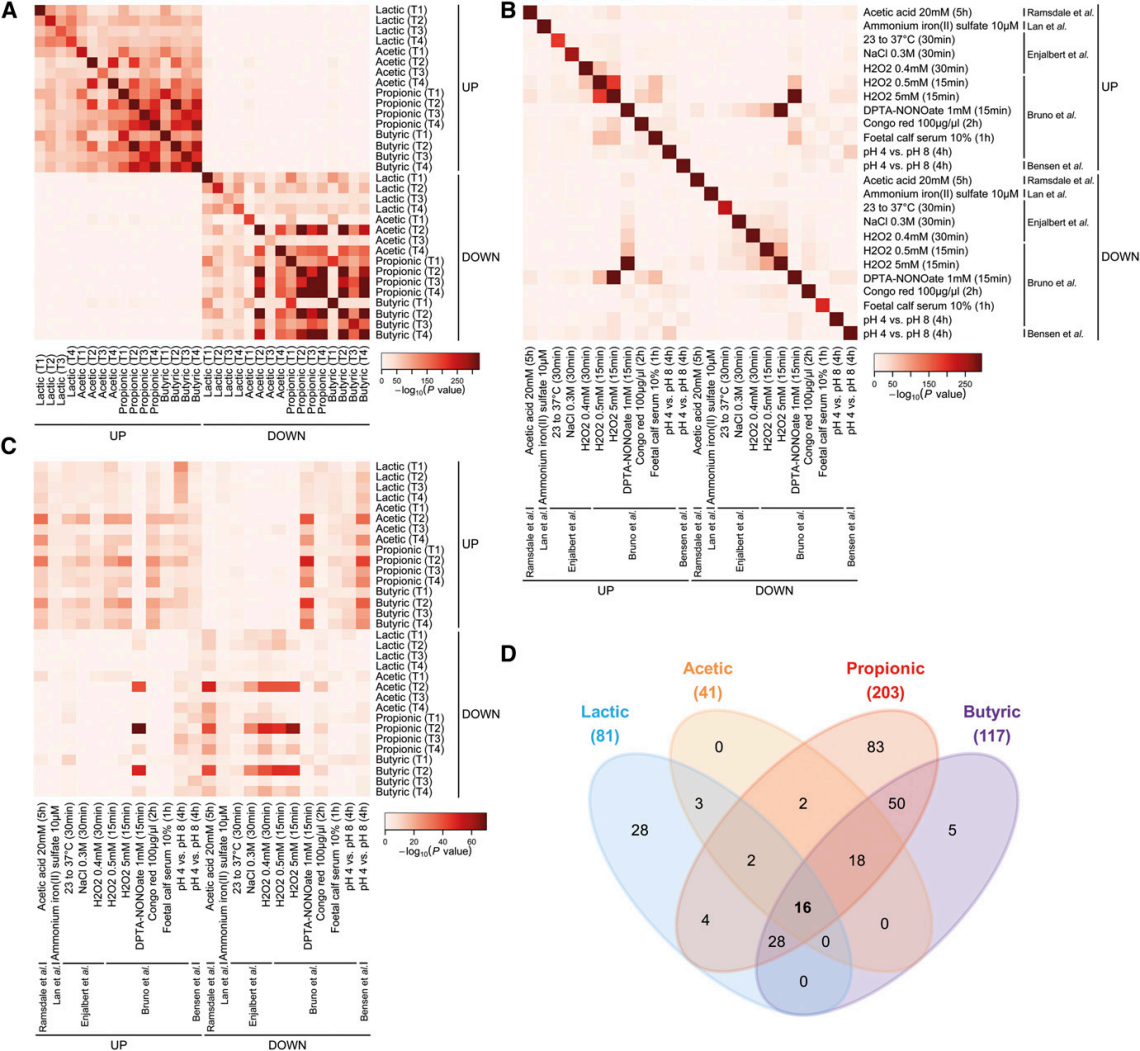
The core transcriptional response of *Candida albicans* to weak organic acids (WOAs): (A−C) Pair-wise overlap analysis between individual lists of genes up- or down-regulated under the indicated conditions. Each heat map is color-coded based on Fisher exact test *P* values between the indicated pair of gene lists according to the color key next to each heat map. Only *P* values for significant overlaps (*i.e.*, odds ratio > 1) are reported. (A) Data relative to lactic, acetic, propionic, and butyric acid are from the present study. (B−C) Gene lists related to acetic acid treatment, iron depletion, heat shock, hyperosmotic stress, oxidative stress, nitrosative stress, Congo red treatment, serum stimulation, and low pH were obtained from previously published work (Enjalbert *et al.* 2003; Bensen *et al.* 2004; Lan *et al.* 2004; Ramsdale *et al.* 2008; Bruno *et al.* 2010) and filtered based on a twofold change threshold. (D) Four-way Venn diagram representing the number of shared and unique genes identified as differentially expressed in each WOA treatment. The figure only reports genes significantly regulated compared to the untreated control (De Man, Rogosa, and Sharpe medium [MRS]) at all four time points, but not in MRS pH 5.5 (power law global error model significance level: 5 × 10^−4^).

To more systematically investigate the similarity between the gene expression profiles induced by the different WOAs at the different time points, we evaluated the degree of overlap between all lists of up- and down-regulated genes obtained in this study (Figure 3A and Figure S4A). By this analysis, we detected highly significant overlaps between different WOA responses, many of which shared >40% of genes in common and some of which as high as >80% (*i.e.*, genes up-regulated by propionic or by butyric acid at T3 and T4). More precisely, genes up-regulated at any given time point in response to any given WOA displayed relatively high overlap with genes up-regulated at the same or at any other time point in response to the same or any other WOA (Figure 3A and Figure S4A). A similar picture was found for genes down-regulated by WOAs.

In an attempt to further annotate these transcriptional responses, we included in this meta-analysis also a series of published lists representing genes differentially expressed (fold change ≥2) in *C. albicans* in response to various kinds of stresses, *i.e.*, heat shock, hyperosmotic stress, oxidative stress, nitrosative stress, treatment with acetic acid or a cell wall perturbing agent, iron depletion, and low pH (Enjalbert *et al.* 2003; Lan *et al.* 2004; Bensen *et al.* 2004; Ramsdale *et al.* 2008; Bruno *et al.* 2010). A few overlaps between previously published stress responses were found; however, most of these overlaps were much lower in both magnitude and statistical significance as compared with overlaps between WOA responses (Figure 3B and Figure S4B). For instance, differentially expressed gene lists obtained under different concentrations of H_2_O_2_ overlapped between each other at most by ~33%, whereas between serum and H_2_O_2_ the overlap was ~20%. Interestingly, genes up-regulated by H_2_O_2_ were significantly enriched in genes down-regulated by a nitric oxide donor, and vice versa, suggesting an opposite transcriptional regulation in response to oxidative and nitrosative stress. In contrast to previous work in *S. cerevisiae* (Gasch *et al.* 2000) and in accordance with a previous report in *C. albicans* (Enjalbert *et al.* 2003), no global resemblance was observed between transcriptional responses of *C. albicans* to different stresses.

When cross-comparing the WOA responses with the previously published stress responses, we found genes up-regulated by the SCFAs acetic, propionic, and butyric to be significantly overlapping with genes down-regulated by nitrosative stress and low pH, most especially at T2 (Figure 3C and Figure S4C). Conversely, genes down-regulated by the same SCFAs at the same time point were enriched in genes up-regulated by nitric oxide and in genes down-regulated by acetic acid, osmotic stress, and oxidative stress. However, both enrichment *p*-values and overlap percentages (all <20%) were far less significant than when comparing WOA responses between each other. Together these results suggest the existence of a stereotypical transcriptional response to WOAs that is largely unrelated to any other previously investigated stress response in *C. albicans*.

Motivated by the relatively high level of similarity between the transcriptional responses of *C. albicans* to individual WOAs at individual time points, we further investigated the overlap between all lists of differentially expressed genes under more stringent conditions, *i.e.*, focusing only on genes consistently and significantly modulated across the entire time course. According to this analysis, 81 genes were found to be consistently modulated by lactic acid at all four time points, whereas 41 were consistently modulated by acetic, 203 by propionic, and 117 by butyric acid (Table S1). When we analyzed the overlap between these gene sets, we found that 83 genes were regulated only by propionic acid, 28 were specifically modulated by lactic acid, and 5 by butyric acid (Figure 3D), indicating that the cellular effect of each of these WOAs might be readily distinguishable from each other on the basis of these gene expression profiles. Among the genes specifically up-regulated by lactic acid, we found a significant enrichment of genes involved in carboxylic acid metabolism, including *CYB2* (orf19.5000), the ortholog of a lactate dehydrogenase gene that was shown to allow *C. glabrata* to assimilate lactate as a sole carbon source (Ueno *et al.* 2011). Although underlain by distinct sets of genes, the 83 propionic-specific genes and the 50 genes commonly regulated by both propionic and butyric acid (but by no other WOA) also were significantly enriched in carboxylic acid metabolism. In addition, the 83 propionic-specific genes were highly enriched in genes involved in translation, ribosome biogenesis, and RNA synthesis, consistent with the previous GO analysis (Figure 2B) that showed these biological functions to be overrepresented among the genes down-regulated by this WOA at all time points, whereas for other WOAs this effect was observed only at some but not all time points. Although no statistically significant enrichment of GO biological functions was found among the five butyric-specific genes, we noticed the presence of the putative ferric reductase *FRP2* (orf19.7112) in this list as well as a significant overrepresentation of iron transporters in the 18 genes specifically regulated by all three SCFAs (acetic, propionic, and butyric), but not by lactic acid, further corroborating the involvement of iron homeostasis in the transcriptional stress response to WOAs. Finally, the 18 SCFA-specific genes also were enriched in genes involved in the response to reactive oxygen species, in accordance with the aforementioned significant overlap with the previously published oxidative stress response genes (Figure 3C).

Despite all the differences between the transcriptional responses induced by each individual WOA, however, this analysis also revealed a core set of 16 genes that were commonly regulated by all WOAs at all times and independently of pH (Figure 3D). Thirteen of these were commonly up-regulated and three commonly down-regulated at all analyzed time points (Figure S5). When we compared this core transcriptional response with that of other published *C. albicans* stress responses, we found the largest similarity with the transcriptional response to reactive oxygen species, with 8 of 13 commonly up-regulated genes and 1 of 3 commonly down-regulated genes (Table S2). Moreover, *CFL2* (orf19.1264), *COI1* (orf19.5063), *FRP1* (orf19.5634), *PIR1* (orf19.220), *FET3* (orf19.4211), *FTR1* (orf19.7219), and *FTR2* (orf19.7231) (all found here to be consistently regulated by all WOAs at all times) were previously reported to be part of the regulatory circuit underlying iron homeostasis (Lan *et al.* 2004; Chen *et al.* 2011). On the opposite spectrum, the heat shock chaperone *HSP90* (orf19.6515), found here as consistently down-regulated by all WOAs at all time-points, was not reported as down-regulated in any other published *C. albicans* stress response study included in our meta-analysis. Quantitative real-time polymerase chain reaction analysis largely confirmed the expression changes identified by RNA-seq for the 16 core WOA stress response genes (File S3 and Table S3). For lactic acid, which was used at a relatively high concentration in the RNA-seq experiment to have a similar growth inhibitory effect as the other WOAs at pH 5.5, these results were further confirmed at a more physiological concentration (64 mM) and at pH 4.5 (Figure S6F). This pH is between that of the gastric and the vaginal mucosa, two prominent niches where lactobacilli normally are found in both mice and humans (Yamaguchi *et al.* 2005; Cribby *et al.* 2008; Walter 2008; O’Hanlon *et al.* 2011).

### WOAs decrease intracellular iron and ribosomal RNA in *C. albicans*

**Figure 4:**
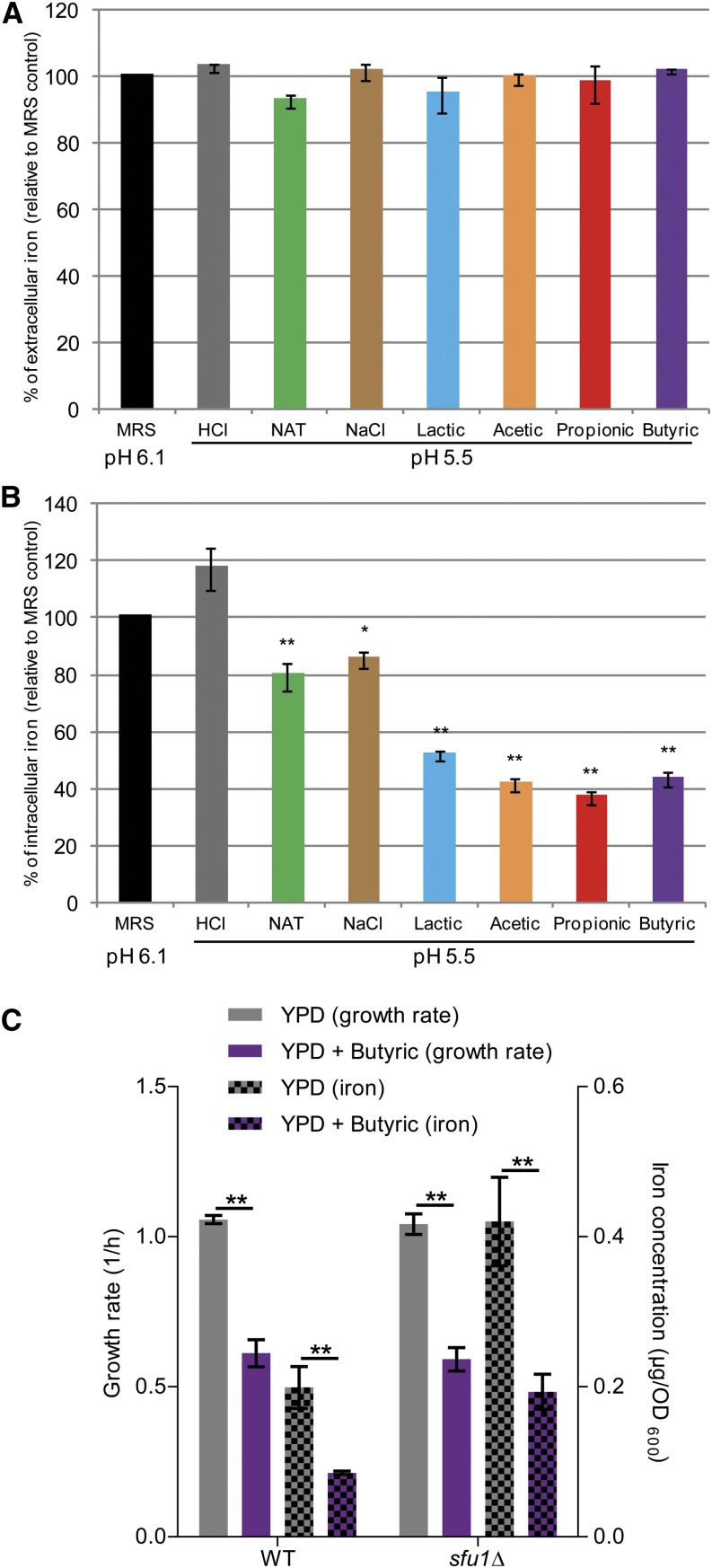
Weak organic acids reduce intracellular iron concentrations in *Candida albicans*: (A−B) *C. albicans* cells were grown under the indicated conditions, and the relative concentration of iron was determined in the culture supernatant (A) or in the cell pellet (B) using a colorimetric assay. MRS, De Man, Rogosa, and Sharpe medium; NAT, nourseothricin. (C) *C. albicans sfu1*Δ mutant and isogenic control (wild type; WT) cells (Noble *et al.* 2010) were grown under the indicated conditions and the relative concentration of iron was determined in the cell pellet using the same colorimetric assay. n = 3; *P < 0.05; **P < 0.01.

Although only a relatively low overall overlap was found between the published list of genes regulated under low iron and all differentially expressed genes found in this study (Figure 3A), iron homeostasis was among the few consistently enriched GO biological processes found across all time points and all WOAs (Figure 2B), and many of the core transcriptional response genes were previously reported to be modulated in low iron conditions in the same direction as our data revealed in response to WOAs (Table S2) (Lan *et al.* 2004; Ramanan and Wang 2000). Based on these findings, we hypothesized that *C. albicans* treated with WOAs might enter a physiological state that, at least in part, resembles the one adopted in a low iron environment. We therefore employed a colorimetric assay (Hsu *et al.* 2011) to quantitatively measure the concentration of iron in both the culture media and supernatants of cells treated with WOAs as well as in cell pellets from the same cultures. Whereas in no case the amount of iron in the extracellular space was found to be limiting for growth (Figure 4A), we surprisingly observed that, as early as 4 hr after incubation with any of the four WOAs the intracellular concentration of iron was reduced by ~60% compared with the untreated cells (Figure 4B). In contrast, in control cultures treated for the same amount of time with HCl to match the same pH (5.5) or with unrelated compounds also known to affect the growth of *C. albicans* to the same extent (*i.e.*, nourseothricin or NaCl), the intracellular iron concentration was only reduced by ~21% or ~15%, respectively. These results show for the first time that WOAs are endowed with the specific effect of reducing intracellular iron concentrations in *C. albicans*. Because WOAs reduced *C. albicans* growth (Figure 1B) (Huang *et al.* 2011), and iron is essential for growth of this species (Ramanan and Wang 2000; Lan *et al.* 2004), we therefore tested whether alteration of intracellular iron concentrations played a role in the inhibition of *C. albicans* growth by WOAs. To this end we used a mutant strain (*sfu1*Δ) previously shown to import more iron than wild type (Chen *et al.* 2011) to test whether counteracting the decreased intracellular iron concentrations would counteract the growth-inhibitory effect of WOAs. As expected, *sfu1*Δ *C. albicans* cells displayed a twofold increase in intracellular iron concentration both in presence and in absence of butyric acid, which brought the intracellular iron concentration of WOA-treated *sfu1*Δ cells to closely resemble the one of untreated wild-type cells (Figure 4C). However, butyric acid had a similar effect on the growth of mutant and wild-type cells, indicating that restoring normal intracellular iron levels is not sufficient to bypass the growth inhibition imposed by WOAs on *C. albicans*.

**Figure 5:**
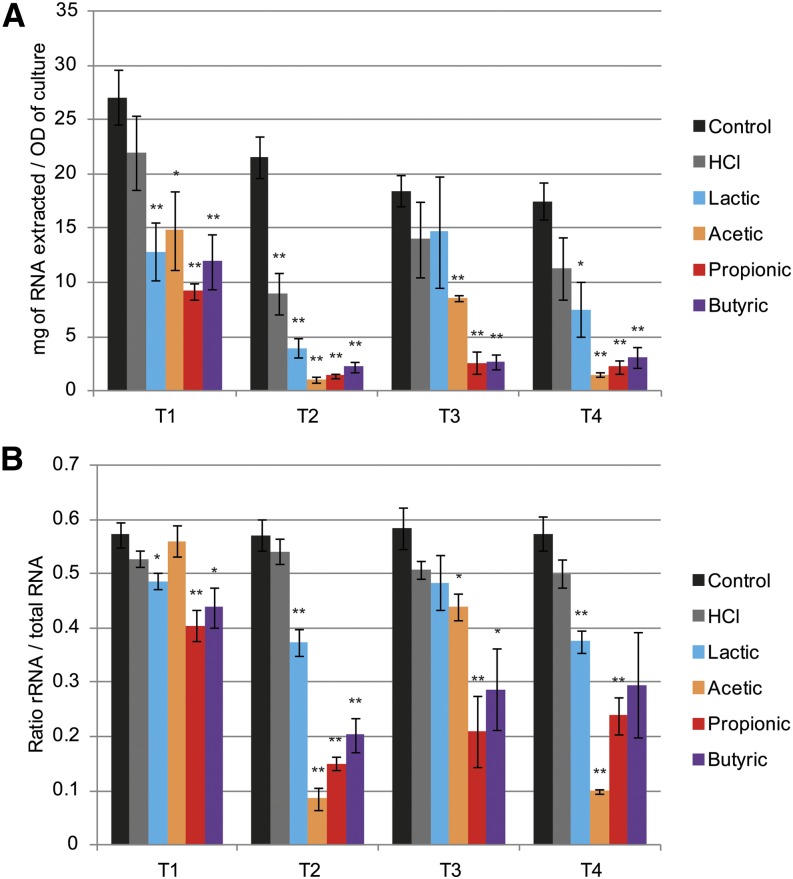
Weak organic acids reduce total RNA and ribosomal RNA in *Candida albicans*: Total RNA content and relative rRNA abundance in cell pellets used for RNA deep-sequencing experiment. (A) Total RNA extraction yield quantified by RiboGreen assay. (B) rRNA *vs.* total RNA ratio estimated by areas under the peaks found on Bioanalyzer electropherograms. n = 4; *P < 0.05; **P < 0.01.

The GO enrichment analysis also revealed a distinctive down-regulation of genes involved in RNA synthesis and ribosome biogenesis (Figure 2B), most especially during chronic exposure to WOAs. In accordance, we noticed during sample preparation a significant decrease in the yield of total RNA extracted per unit biomass between WOA-treated and -untreated samples (~50%) as well as between late and early time points (up to ~90%) (Figure 5A). Moreover, WOA treatment caused a significant reduction in the relative abundance of ribosomal RNA over total RNA (Figure 5B). These data suggest that prolonged exposure of *C. albicans* to WOAs leads to a general down-regulation of both transcription and translation.

## Discussion

In the present study, we comprehensively investigated the effect of WOAs on the transcriptome of the opportunistic fungal pathogen *C. albicans*. WOAs represent the most abundant group of microbiota-derived metabolites found on human body surfaces, where *C. albicans* is thought to reside as a benign commensal. However, with the exception of a few studies (Noverr and Huffnagle 2004; Ramsdale *et al.* 2008; Huang *et al.* 2011; Kohler *et al.* 2012), little was known about the effects of the WOAs lactic, acetic, propionic, and butyric acid on *C. albicans*.

Our unbiased transcriptomic approach revealed both commonalities and differences in the global transcriptional response of *C. albicans* to lactic, acetic, propionic, and butyric acid. Despite marked differences between acids and between time points, we were nonetheless successful in identifying a pH-independent core transcriptional response to all four tested acids at all times. Two possible scenarios can explain this apparent contradiction. Although each WOA might interact with its own unique cellular target, effects downstream of these interactions might partially converge on a shared set of genes. Alternatively, each WOA might interact with more than one target, some of which might be shared between acids. Although further research is required to fully understand the mechanism of action of each of these microbiota-derived metabolites on *C. albicans* biology, these results clearly suggest that the effect of WOAs cannot simply be attributed to a response to acidification. In fact, we found little or no overlap between our lists of differentially expressed genes and the genes previously reported to respond to low pH (Figure 3C and Figure S4C) (Bensen *et al.* 2004). Moreover, although we found up-regulation of iron acquisition genes as part of the core response conserved across WOAs, a similar transcriptional response was previously reported to occur under alkaline, as opposed to acidic, conditions (Bensen *et al.* 2004). Overall, our data are consistent with previous conclusions that *C. albicans* does not possess a general transcriptional response to stress (Enjalbert *et al.* 2003), unlike in the other yeast species *Saccharomyces cerevisiae* and *Schizosaccharomyces pombe*, where a stereotypical gene expression modulation termed the “environmental stress response” was found to be consistently engaged in response to a large variety of different stresses (Gasch *et al.* 2000; Chen *et al.* 2003). Nevertheless, and most probably because of the chemical and structural similarity between the herein-tested WOAs, we were able to identify a set of 16 genes commonly regulated by all WOAs at all times, which only partially overlapped with a few previously studied stress response in *C. albicans*. We thus hypothesize these 16 genes to play an important role in the physiological response of *C. albicans* to WOAs.

The global transcriptional response to WOAs was significantly enriched of genes involved in iron homeostasis; host interaction; glycolysis; ATP, ergosterol, arginine, and RNA biosynthesis; and ribosome biogenesis. In contrast, the common response to the WOAs acetic, propionic, and sorbic acid in *S. cerevisiae* was underlain by a different set of genes involved in protein folding, lipid metabolism, cell wall function, and multidrug resistance (Mira *et al.* 2010b). In addition to species-specific differences, we noted that the *S. cerevisiae* experiment was performed at pH ~4, which is below the acid dissociation constant (p*K*_a_) of these acids, whereas in our experiment the pH (5.5) was above the p*K*_a_. However, the influence of this parameter on the resulting gene expression profiles remains to be established. Interestingly, another report showed that at pH 4.5 several genes associated with iron uptake were identified to provide tolerance to acetic acid in *S. cerevisiae* (Mira *et al.* 2010a). In accordance with our observations, iron uptake therefore appears to play an important role in the fungal response to WOAs. 

Furthermore, chelation of extracellular iron induces expression of iron transporters in *C. albicans* (Lan *et al.* 2004) in a manner similar to what we report here in response to WOAs. In our settings, however, cells were not facing a limiting availability of extracellular iron (Figure 4A) but were depleted of iron in the intracellular compartment (Figure 4B). This finding was unexpected, and it still remains to be elucidated whether this was due to an inhibition of iron import or a stimulation of its export. However, a mutant strain altered in iron uptake such as *sfu1*Δ (Chen *et al.* 2011), which displayed the expected increase in intracellular iron concentration, showed a similar sensitivity to butyric acid as the wild type (Figure 4C). Although a promising candidate mechanism, decreased intracellular iron concentrations are hence unlikely to be the cause of the growth-inhibitory effect of WOAs. Nevertheless, iron uptake is critical for several different aspects of *C. albicans* physiology, including competition with the microflora and interaction with the host (Ramanan and Wang 2000; Purschke *et al.* 2012; Perez *et al.* 2013), so the fact that microbiota-derived WOAs impinge on the intracellular availability of this essential micronutrient is likely to be of great biological importance and deserves further investigation.

The second major pathway regulated in response to WOA treatment was a significant down-regulation of RNA synthesis and ribosome biogenesis genes, which was mostly apparent upon chronic exposure. Such a reduction in RNA synthesis has been reported previously during exposure to stress in *S. cerevisiae* (Lempiainen and Shore 2009). In accordance, stress can neutralize the TORC1 complex (Takahara and Maeda 2012), which plays an important role in RNA synthesis (Lempiainen and Shore 2009). It remains to be evaluated whether in *C. albicans* WOAs directly activate this pathway or whether the observed total RNA reduction was an indirect consequence of a WOA-mediated stress. Furthermore, WOA treatment caused a significant reduction in the rRNA to total RNA ratio (Figure 5B). This observation is reminiscent of a stereotypical stress response shared across different microbial species, in which the number of ribosomes per cell is tightly regulated to match the growth rate of the cells (Lempiainen and Shore 2009). Because ribosomal RNA is well known to represent the majority of the total RNA in a typical cell (Kampers *et al.* 1996), this result could also explain the overall reduction in total RNA observed after WOA treatment.

Overall, our RNA-sequencing data unveiled a multifaceted picture of the transcriptional response of *C. albicans* to WOAs, with several genes and pathways uniquely modulated by each molecule, but also a robust core stress response that might serve as a signature of the exposure of this fungal species to lactic, acetic, propionic, or butyric acid. Furthermore, these data indicate that exposure of *C. albicans* to WOAs over prolonged periods of time, a situation likely to arise in its natural niche when co-inhabiting the human body with large numbers of WOA-producing bacteria, might progressively shift the cells into a “starvation-like” metabolic state, characterized by low rates of transcription, translation, and growth. Future research is warranted to elucidate how this altered physiological state might affect interaction of this fungus with the rest of the microbiota as well as with its host.

## 

## Supplementary Material

Supporting Information
