# Supplementary material for: The Transcriptional Stress Response of Candida albicans to Weak Organic Acids
Source: G3 (Bethesda). 2015 Jan 29;5(4):497–505. doi: 10.1534/g3.114.015941 (PMC4390566; doi:10.1534/g3.114.015941)
Supplement: Supporting Information [file supp_5_4_497__index.html]

The Transcriptional Stress Response of Candida albicans to Weak Organic Acids — Supporting Information 

# The Transcriptional Stress Response of *Candida albicans* to Weak Organic Acids

## Supporting Information for Cottier *et al.*, 2015

**Files in this Data Supplement:**

- Supporting Information - Figures S1-S6, Tables S1-S3, Files S1-S3, and References (PDF, 1 MB)
- Figure S1 - No evidence for aneuploidy in any sequenced sample. (PDF, 475 KB)
- Figure S2 - Global transcriptional response of *C. albicans* to WOAs. (PDF, 595 KB)
- Figure S3 - PLGEM goodness-of-fit. (PDF, 648 KB)
- Figure S4 - Comparison of *C. albicans* transcriptional response to WOA and other transcriptional stress responses. (PDF, 441 KB)
- Figure S5 - Expression levels of the 16 core genes commonly regulated by all WOAs at all times. (PDF, 488 KB)
- Figure S6 - Quantitative RT-PCR validates gene regulation observed by RNA-sequencing. (PDF, 307 KB)
- Table S1 - Genes significantly regulated in response to each WOA at all time points. (PDF, 120 KB)
- Table S2 - Comparison of the 16 WOA core response genes to previously published *C. albicans* transcriptional stress responses. (PDF, 162 KB)
- Table S3 - Quantitative RT-PCR primers used in this study. (PDF, 119 KB)
- File S3 - Additional Materials and Methods (PDF, 120 KB)
- File S1 - Fold changes and p-values of each transcript in every condition. Each average fold change was calculated as the ratio of the mean normalized RPKM value in the treated condition over the mean normalized RPKM value in the time-matched untreated condition. P-values represent significance of differential expression according to a PLGEM analysis (Pavelka et al. 2004). (.xlsx, 3 MB)
- File S2 - GO enrichment in genes regulated in the different conditions. Enrichment of GO Biological Process terms was performed on all lists of genes significantly regulated at any time point (T1, T2, T3 or T4) in response to lactic, acetic, propionic or butyric acid, following removal of genes significantly regulated by HCl. Values correspond to multiple-testing corrected p-values obtained from CGD (Inglis et al. 2012). Only p-value ≤ 0.1 are reported. Positive values refer to enrichment within the up-regulated genes, while negative values indicate enrichment among the down-regulated genes. (.xlsx, 55 KB)
