# Supplementary material for: The Transcriptional Stress Response of Candida albicans to Weak Organic Acids
Source: G3 (Bethesda). 2015 Jan 29;5(4):497–505. doi: 10.1534/g3.114.015941 (PMC4390566; doi:10.1534/g3.114.015941)
Supplement: Supporting Information [file supp_g3.114.015941_015941SI.pdf]

G3 · Genes | Genomes | Genetics

The Transcriptional Stress Response of *Candida albicans* to Weak Organic Acids

Fabien Cottier[*], Alrina Shin Min Tan[*], Jinmiao Chen[*], Josephine Lum[*], Francesca Zolezzi[*], Michael Poidinger[*,§], Norman Pavelka[*,†,1]

[*] Singapore Immunology Network (SIgN), Agency for Science, Technology and Research (A*STAR), Singapore 138648, Singapore
[§] Department of Biological Sciences, National University of Singapore, Singapore 117543, Singapore
[†] School of Biological Sciences, Nanyang Technological University, Singapore 637551, Singapore

[1] Corresponding author: Singapore Immunology Network (SIgN), Agency for Science, Technology and Research (A*STAR), 8A Biomedical Grove, Immunos #04, Singapore 138648, e-mail: norman_pavelka@immunol.a-star.edu.sg

DOI: 10.1534/g3.114.015941

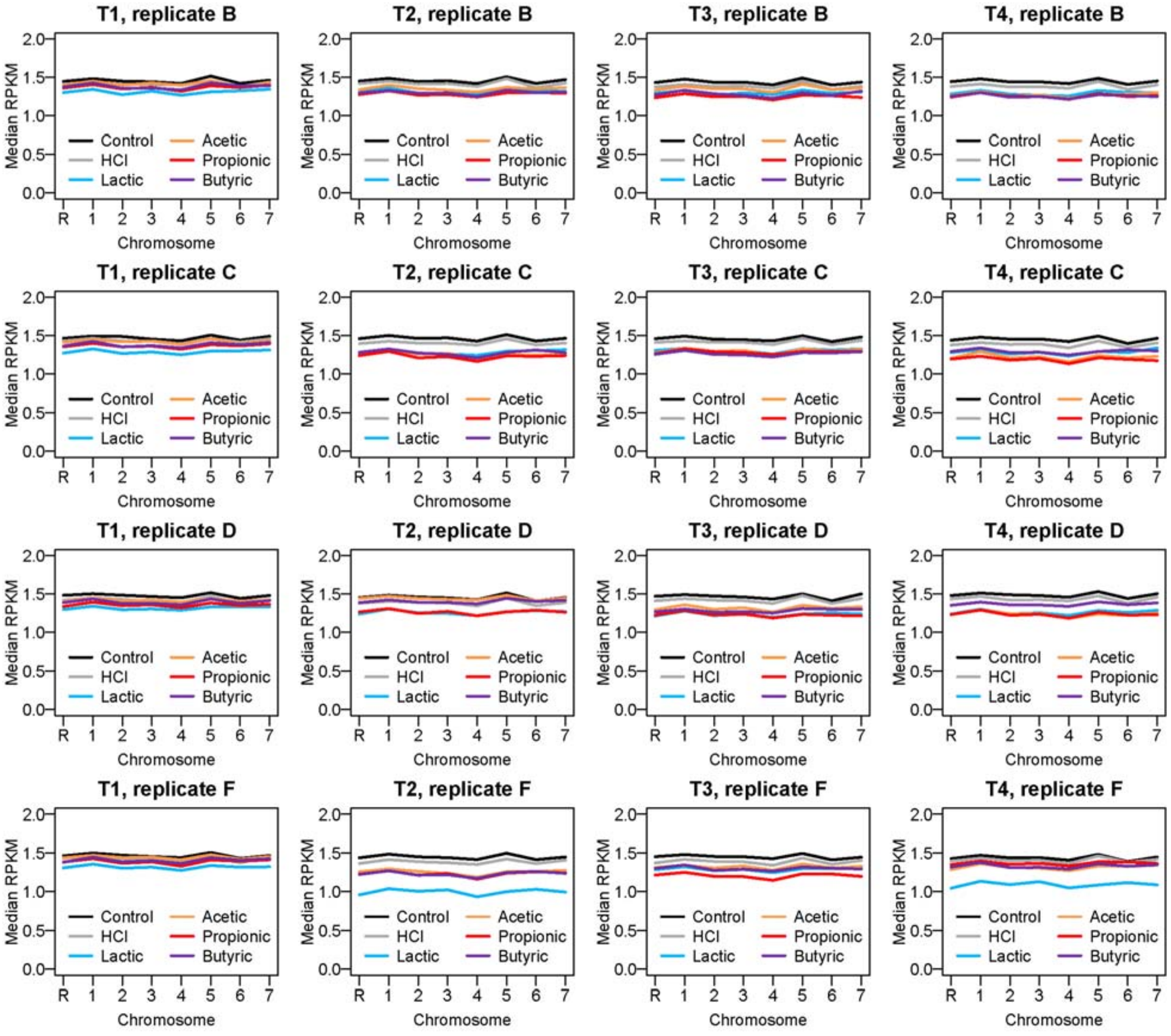

Figure S1 No evidence for aneuploidy in any sequenced sample. The median expression value (log10-transformed RPKM) of each of the eight chromosomes in the *C. albicans* genome is plotted for each of the 96 sequenced transcriptome samples. Samples are grouped by time point (column) and replicate (row). None of the samples displayed observable deviations from uniformity between the chromosomes, which would have otherwise indicated a possible presence of aneuploidy.

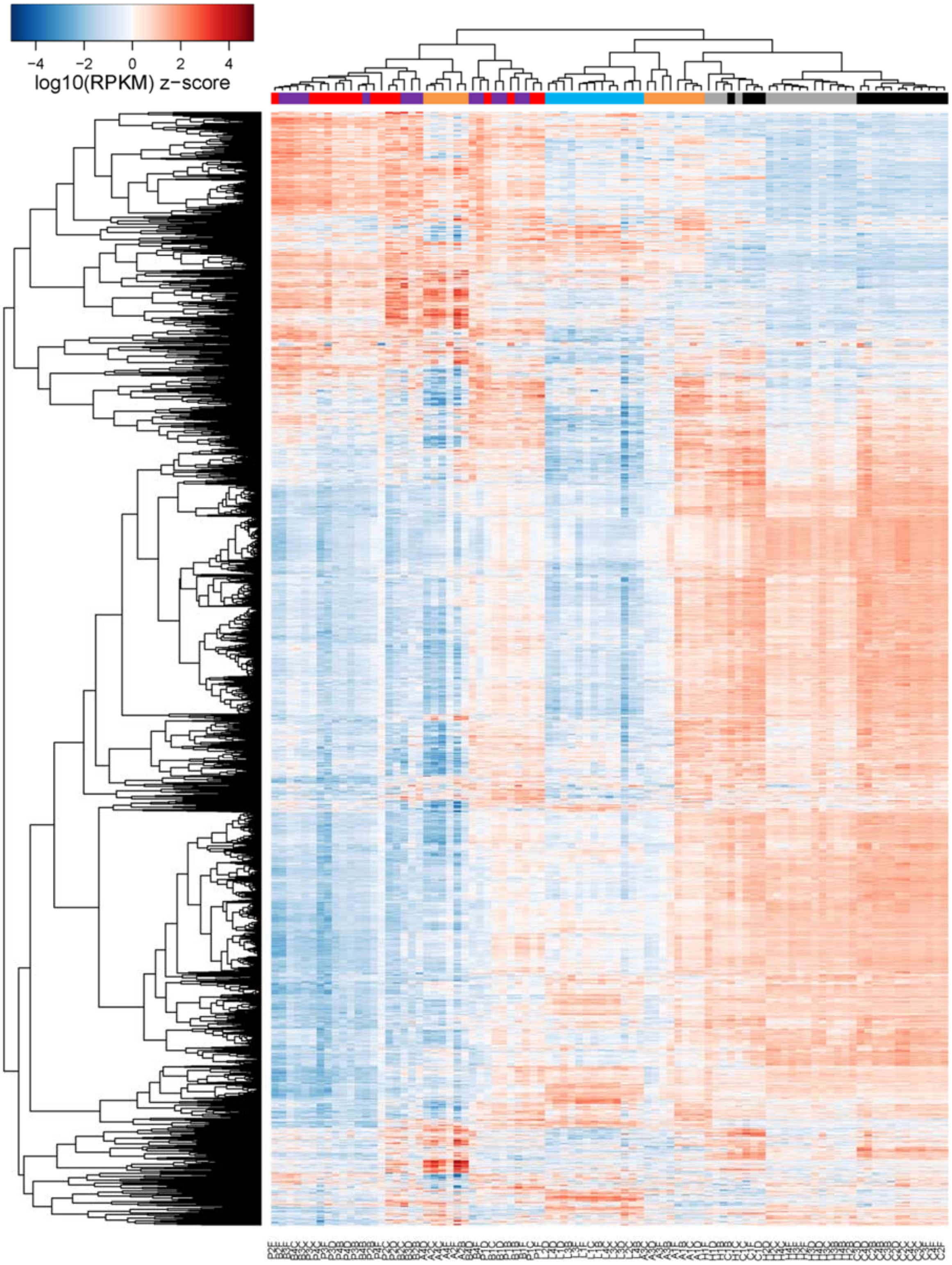

Figure S2 Global transcriptional response of *C. albicans* to WOAs. The figure displays a two-way hierarchical clustering of 6,453 transcripts (rows) and 89 samples (columns). Sample names are indicated at the bottom and follow the same nomenclature described in the legend of Figure 2. Samples labeled as L2F, L4F, L2B, A4B, B2D, A2D and B3B were discarded as outliers from this and all other analyses. Log10-transformed RPKM expression values were converted to z-scores, with red indicating expression levels above and blue symbolizing expression levels below the mean expression level of each gene across the samples. Distance metric = 1 − Pearson correlation.

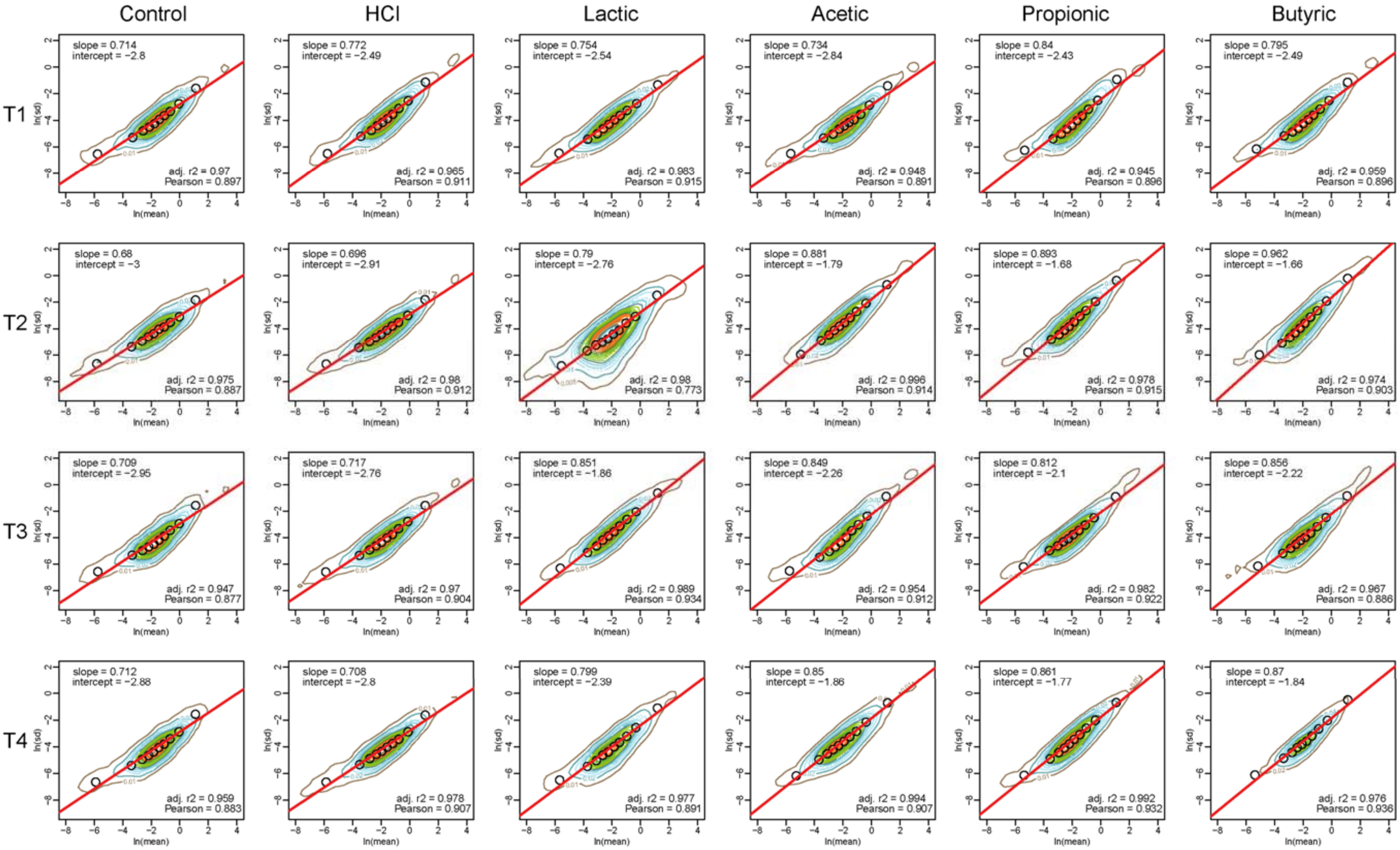

Figure S3 PLGEM goodness-of-fit. A PLGEM was fitted to each set of biological replicates corresponding to an individual treatment (column) and time point (row). For each set of replicates, the logarithm of the standard deviation of the normalized RPKM values [ln(sd)] was plotted against the logarithm of the mean normalized RPKM value [ln(mean)] calculated for each of 6,453 transcripts. Diagnostic plots of the goodness-of-fit include a contour plot of all analyzed transcripts, ten modeling points (black circles) and a linear regression representing the PLGEM (red line). Inset numbers in each plot represent the slope, the intercept and the adjusted r^2 of the linear regression based on the modeling points, as well as the Pearson correlation of all ln(sd) vs. ln(mean) values. Details of the PLGEM algorithm and a definition and interpretation of all above parameters have been previously described (Pavelka et al. 2004).

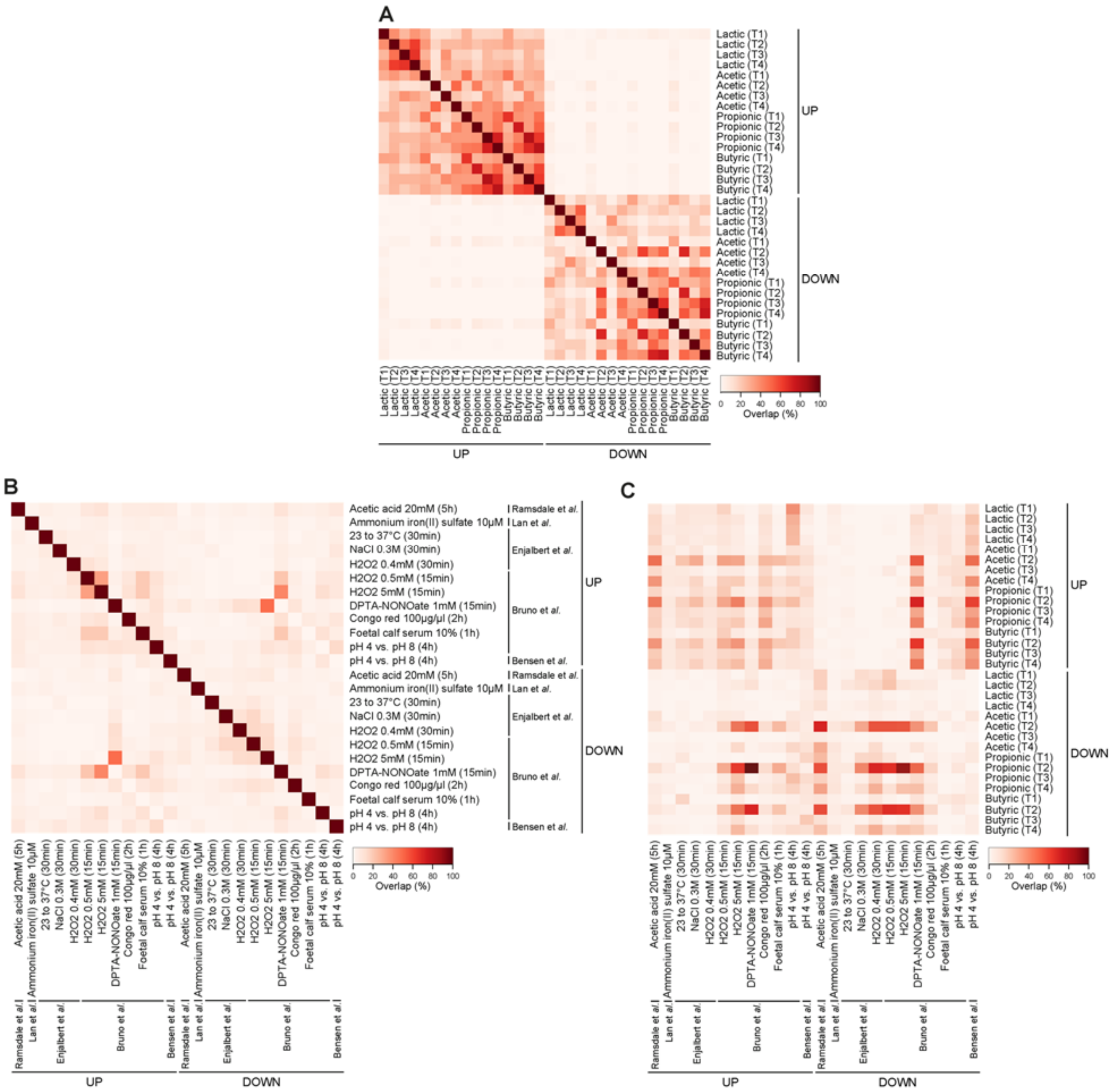

Figure S4 Comparison of *C. albicans* transcriptional response to WOA and other transcriptional stress responses. (A-C) Pair-wise overlap analysis between individual lists of genes up- or down-regulated under the indicated conditions. Each heat map is color-coded based on the percentage of overlap between the indicated pair of gene lists according to the color key next to each heat map. Samples are identical to the ones presented on Figure 3.

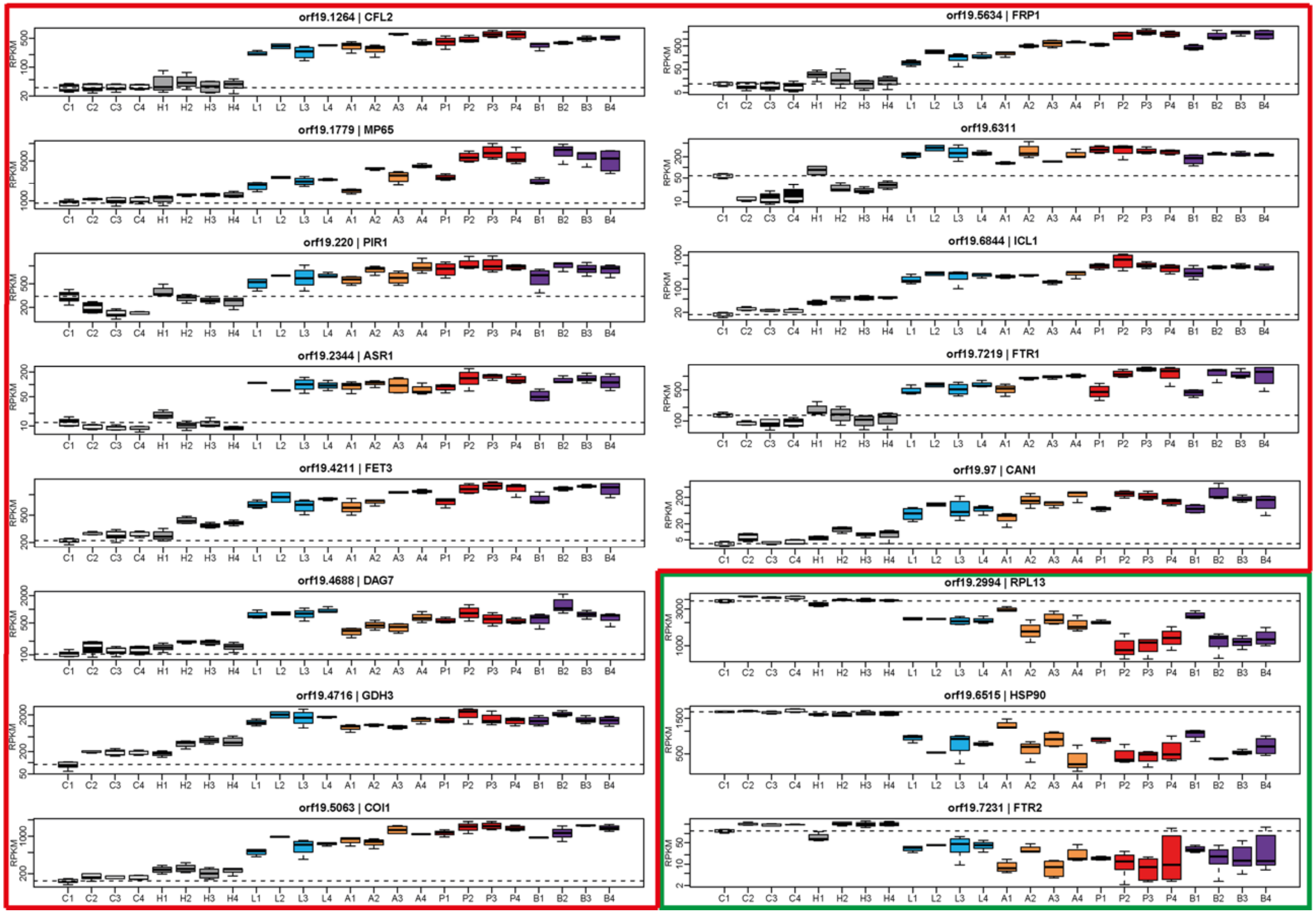

Figure S5 Expression levels of the 16 core genes commonly regulated by all WOAs at all times. Genes included in the red frame were up-regulated in presence of all WOAs throughout the time course, while the genes framed in green were consistently down-regulated. Data are represented as boxplots to show the distribution across the biological replicates.

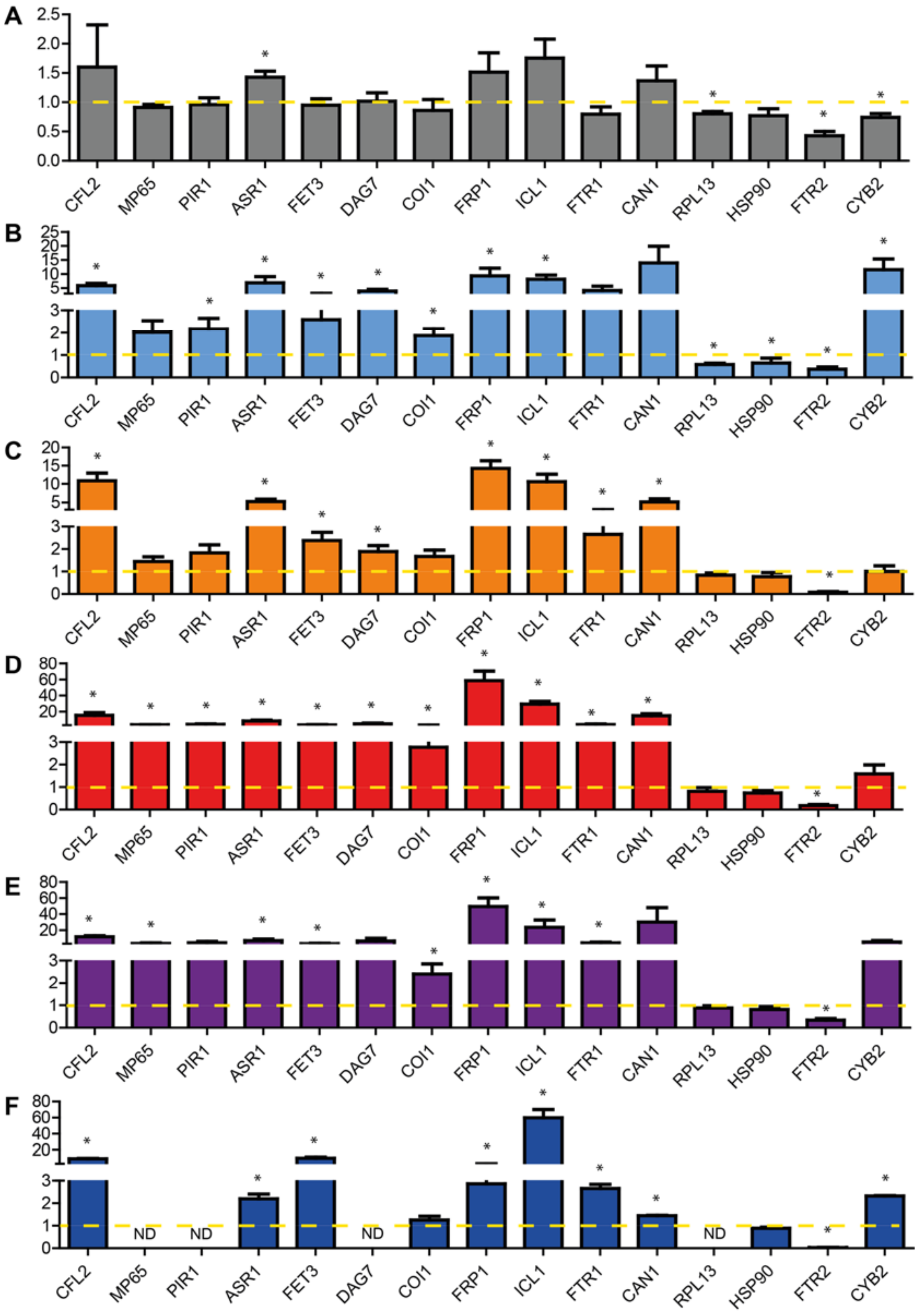

Figure S6 Quantitative RT-PCR validates gene regulation observed by RNA-sequencing. Relative expression levels of the indicated genes were normalized to the *RIP1* endogenous control to confirm significant regulation during exposure to HCl (A), lactic (B), acetic (C), propionic (D) and butyric acid (E) at pH 5.5 compared to untreated control cultures (MRS). (A-E) qPCR performed on same cDNA used for RNA-seq. (F) Similar experiment under exposure to lactic acid (62 mM) at pH 4.5 compared to the control (MRS). Statistical analysis was performed in Microsoft Excel, using one-sample t-test assuming equal variances. $n \geq 3$; * $p < 0.05$

Table S1 Genes significantly regulated in response to each WOA at all time points. Genes differentially expressed in response to lactic, acetic, propionic or butyric acid at T1, T2, T3 and T4. These lists were used to draw the Venn diagram in Figure 3D. Transcript identifiers refer to the *C. albicans* Assembly 21 genome annotation.

**Weak organic acid**	**Genes significantly regulated in the same direction at all time-points**
Lactic	orf19.1075.1, orf19.125, orf19.1264, orf19.1358, orf19.1480, orf19.1779, orf19.1857, orf19.2021, orf19.2066.1, orf19.220, orf19.2344, orf19.251, orf19.2525, orf19.2765, orf19.3160, orf19.3171, orf19.3340, orf19.3548.1, orf19.3954.1, orf19.4211, orf19.4216, orf19.4506, orf19.4551, orf19.4688, orf19.4716, orf19.5000, orf19.5005, orf19.5032, orf19.5063, orf19.5213.1, orf19.5517, orf19.5610, orf19.5634, orf19.5653, orf19.5753, orf19.5805, orf19.6165, orf19.6311, orf19.6724, orf19.6757, orf19.6844, orf19.6854, orf19.691, orf19.7219, orf19.7279.1, orf19.7398.1, orf19.7417, orf19.7469, orf19.789, orf19.97, ITS2, orf19.1030, orf19.1389, orf19.1697, orf19.200, orf19.2111.2, orf19.2767, orf19.2994, orf19.3572.3, orf19.3810, orf19.4054, orf19.4336, orf19.4492, orf19.4623, orf19.4660, orf19.4909.1, orf19.493, orf19.5225.2,orf19.5351, orf19.5904, orf19.5943.1, orf19.5964.2, orf19.6220.4, orf19.6414.3, orf19.6515, orf19.7018, orf19.7046, orf19.7231, orf19.7477 ,orf19.827.1, orf19.962
Acetic	orf19.1263, orf19.1264, orf19.1395, orf19.1779, orf19.2062, orf19.220, orf19.2344, orf19.2762, orf19.3160, orf19.3461, orf19.3548.1, orf19.4211, orf19.4216, orf19.4505, orf19.4688, orf19.4716, orf19.4802, orf19.5063, orf19.5634, orf19.5952, orf19.6073, orf19.6116, orf19.6311, orf19.6844, orf19.7021, orf19.7111.1, orf19.7219, orf19.734, orf19.97, orf19.1065, orf19.1517, orf19.238, orf19.2767, orf19.2994, orf19.3433, orf19.386, orf19.4674.1, orf19.6515, orf19.6948, orf19.7046, orf19.7231
Propionic	orf19.1030, orf19.1065, orf19.1154, orf19.1470, orf19.1517, orf19.1601, orf19.1697, orf19.1700, orf19.1770, orf19.1853, orf19.1996, orf19.2067, orf19.2091, orf19.2111.2, orf19.2179.2, orf19.2232, orf19.2310.1, orf19.2329.1, orf19.236, orf19.238, orf19.2422, orf19.2451, orf19.2478.1, orf19.269, orf19.2709, orf19.2767, orf19.2859, orf19.2864.1, orf19.2871, orf19.2935, orf19.2948, orf19.2994, orf19.3002, orf19.3034, orf19.3087, orf19.3138, orf19.3175, orf19.3325.3, orf19.336, orf19.3396, orf19.3415.1, orf19.3426, orf19.3433, orf19.3572.3, orf19.3788.1, orf19.3789, orf19.3810, orf19.3812, orf19.385, orf19.386, orf19.3870, orf19.3962, orf19.4026, orf19.4040, orf19.4060, orf19.4093, orf19.4336, orf19.4375.1, orf19.4447, orf19.4468, orf19.4490, orf19.4492, orf19.4622, orf19.4623, orf19.4660, orf19.4674.1, orf19.4758, orf19.4909.1, orf19.493, orf19.5077, orf19.5126, orf19.5225.2, orf19.526, orf19.5341, orf19.5351, orf19.5466, orf19.5750, orf19.5838, orf19.5858, orf19.5904, orf19.5943.1, orf19.5949, orf19.5964.2, orf19.6002, orf19.6085, orf19.6090, orf19.6220.4, orf19.6253, orf19.6264.4, orf19.6265, orf19.6286.2, orf19.6375, orf19.637, orf19.6515, orf19.6541, orf19.6632, orf19.6702, orf19.6749, orf19.6785, orf19.687.1, orf19.6948, orf19.7018, orf19.7046, orf19.7048.1, orf19.7050, orf19.7161, orf19.7217, orf19.7231, orf19.7236, orf19.7397.1, orf19.7424, orf19.7477, orf19.7654, orf19.827.1, orf19.838.1, orf19.1075.1, orf19.1149, orf19.1227, orf19.1237, orf19.125, orf19.1263, orf19.1264, orf19.1339, orf19.1395, orf19.1690, orf19.1779, orf19.1891, orf19.1974, orf19.2062, orf19.2125, orf19.2158, orf19.2179, orf19.220, orf19.2344, orf19.23, orf19.2608, orf19.2686, orf19.2762, orf19.2990, orf19.3053, orf19.3104, orf19.3234, orf19.3340, orf19.3378, orf19.3461, orf19.3507, orf19.3538, orf19.3612, orf19.36.1, orf19.3732, orf19.4082, orf19.4211, orf19.4505, orf19.4565, orf19.4630, orf19.4688, orf19.4716, orf19.4802, orf19.4833, orf19.4886, orf19.4905, orf19.5024, orf19.5063, orf19.5112, orf19.5379, orf19.5517, orf19.5610, orf19.5634, orf19.5653, orf19.5683, orf19.5686, orf19.5730, orf19.5777, orf19.5791, orf19.5801, orf19.5805, orf19.5952, orf19.6073, orf19.6116, orf19.6311, orf19.6318, orf19.638, orf19.6840, orf19.6844, orf19.6854, orf19.6877, orf19.6951, orf19.7021, orf19.7111.1, orf19.7196, orf19.7219, orf19.7279.1, orf19.7306, orf19.734, orf19.7398.1, orf19.7417, orf19.7469, orf19.7531, orf19.7611, orf19.7676, orf19.767, orf19.822, orf19.97
Butyric	orf19.1030, orf19.1065, orf19.1517, orf19.1601, orf19.1697, orf19.2111.2, orf19.238, orf19.2451, orf19.2864.1, orf19.2994, orf19.3002, orf19.3175, orf19.3572.3, orf19.3810, orf19.385, orf19.386, orf19.4040, orf19.4336, orf19.4447, orf19.4660, orf19.4674.1, orf19.4909.1, orf19.493, orf19.5225.2, orf19.5466, orf19.5750, orf19.5858, orf19.5904, orf19.5943.1, orf19.5964.2, orf19.6090, orf19.6220.4, orf19.6515, orf19.6541, orf19.6632, orf19.687.1, orf19.6948, orf19.7018, orf19.7231, orf19.827.1, orf19.838.1, orf19.1048, orf19.1075.1, orf19.1149, orf19.1227, orf19.1237, orf19.125, orf19.1263, orf19.1264, orf19.1339, orf19.1395, orf19.1690, orf19.1779, orf19.1891, orf19.1974, orf19.2062, orf19.2158, orf19.2160, orf19.220, orf19.2344, orf19.2762, orf19.3104, orf19.3234, orf19.3340, orf19.3378, orf19.3461, orf19.3507, orf19.3612, orf19.36.1, orf19.4211, orf19.4505, orf19.4565, orf19.4609, orf19.4688, orf19.4716, orf19.4773, orf19.4802, orf19.4833, orf19.4886, orf19.4905, orf19.5063, orf19.5112, orf19.5379, orf19.5517, orf19.5610, orf19.5634, orf19.5653, orf19.5683, orf19.5686, orf19.5730, orf19.5791, orf19.5801, orf19.5805, orf19.5952, orf19.6073, orf19.6116, orf19.6311, orf19.6318, orf19.638, orf19.6844, orf19.6854, orf19.6877, orf19.6951, orf19.7111.1, orf19.7112, orf19.7196, orf19.7219, orf19.7279.1, orf19.7306, orf19.734, orf19.7398.1, orf19.7417, orf19.7469, orf19.7531, orf19.7611, orf19.822, orf19.97

Table S2 Comparison of the 16 WOA core response genes to previously published *C. albicans* transcriptional stress responses. The presence of each of the 16 core response genes to WOAs (13 up-regulated and 3 down-regulated genes) in previously published gene lists was evaluated. A bullet symbolizes that the indicated WOA core response transcript was found to be at least two-fold up-regulated (A) or down-regulated (B) in the previously published *C. albicans* transcriptional stress response indicated in the corresponding column header. Genes consistently up-regulated (A) or consistently down-regulated (B) in both gene lists are highlighted in pink (A) or light blue (B), respectively.

(A) Genes up-regulated in previously published *C. albicans* stress responses.

**WOA core response**			**Acetic acid 20mM (5h)**	**Ammonium iron(II) sulfate 10µM**	**23 to 37 °C (30min)**	**NaCl 0.3M (30min)**	**H_2O_2 0.4mM (30min)**	**H_2O_2 0.5mM (15min)**	**H_2O_2 5mM (15min)**	**DPTA-NONOate 1mM (15min)**	**Congo red 100µg/µl (2h)**	**Foetal calf serum 10% (1h)**	**pH 4 vs. pH 8 (4h) [Bruno et al.]**	**pH 4 vs. pH 8 (4h) [Bensen et al.]**
UP	orf19.1264	CFL2		•				•		•				•
	orf19.1779	MP65												•
	orf19.220	PIR1	•											
	orf19.2344	ASR1			•	•		•	•		•	•	•	
	orf19.4211	FET3								•				
	orf19.4688	DAG7				•				•			•	
	orf19.4716	GDH3						•						
	orf19.5063	COI1		•							•			
	orf19.5634	FRP1		•				•			•	•		
	orf19.6311	orf19.6311	•		•			•	•	•				
	orf19.6844	ICL1	•					•	•				•	
	orf19.7219	FTR1						•		•	•			
	orf19.97	CAN1	•				•	•			•		•	
DOWN	orf19.2994	RPL13												•
	orf19.6515	HSP90												
	orf19.7231	FTR2								•				•

(B) Genes down-regulated in previously published *C. albicans* stress responses.

WOA core response			Acetic acid 20mM (5h)	Ammonium iron(II) sulfate 10µM	23 to 37 °C (30min)	NaCl 0.3M (30min)	H_2O_2 0.4mM (30min)	H_2O_2 0.5mM (15min)	H_2O_2 5mM (15min)	DPTA-NONOate 1mM (15min)	Congo red 100µg/µl (2h)	Foetal calf serum 10% (1h)	pH 4 vs. pH 8 (4h) [Bruno et al.]	pH 4 vs. pH 8 (4h) [Bensen et al.]
UP	orf19.1264	CFL2											•	
	orf19.1779	MP65							•					
	orf19.220	PIR1		•				•				•		
	orf19.2344	ASR1												•
	orf19.4211	FET3			•	•			•		•	•	•	
	orf19.4688	DAG7			•			•	•		•			
	orf19.4716	GDH3				•			•	•		•	•	
	orf19.5063	COI1												
	orf19.5634	FRP1							•	•			•	
	orf19.6311	orf19.6311												
	orf19.6844	ICL1								•				
	orf19.7219	FTR1							•					
	orf19.97	CAN1												
DOWN	orf19.2994	RPL13	•											
	orf19.6515	HSP90												
	orf19.7231	FTR2		•	•		•	•	•		•			

Table S3 Quantitative RT-PCR primers used in this study.

Primer name	Sequence
ASR1-RT-F	ATGGCGATTCCAGTTATGGT
ASR1-RT-R	GCATCAAGATGGTCAACACC
CAN1-RT-F	CAAGCTGGTGTTGCTCCTAA
CAN1-RT-R	TGACTGGAACAGGCCAAGTA
CFL2-RT-F	TATCGAAGGACGTCCAAACA
CFL2-RT-R	AGGATGTCCACACGTAACGA
COI1-RT-F	TGGATGTTATGAAACAACAAGATG
COI1-RT-R	ATTTAGCACAAGCACCACCA
CYB2-RT-F	TACGGTTGATGCTCCACAAT
CYB2-RT-R	AAATGGCTCTTGCTGAACCT
DAG7-RT-F	CTCCATCAACAACTTCAGCAA
DAG7-RT-R	CAACAATGAAATCGGAATCG
FET3-RT-F	GCCATATTGAATGGCATTTG
FET3-RT-R	GGCATTACCTTCCCAGGATA
FRP1-RT-F	TTTGGTGCAACACCGTATTT
FRP1-RT-R	AACCCTGTCGATAACCAACC
FTR1-RT-F	ACTGGTGGTGATGCTTCTGA
FTR1-RT-R	CCGTTATCGGTTTCTGGATT
FTR2-RT-F	AATTTGTGGTCTTGCAGTGG
FTR2-RT-R	CTCTGGAGAACAAACCAGCA
HSP90-RT-F	TACGAACCATTGACCAAAGC
HSP90-RT-R	CAGACCAACCAAATTGACCA
ICL1-RT-F	AGAAATTGGCCAAGGAATTG
ICL1-RT-R	GACGGCACATTGAGTACCAC
MP65-RT-F	AACTCTGGTGCTTGGGTCTT
MP65-RT-R	TAGATGGCCAACCAGTTTCA
PIR1-RT-F	GCTGCTGCTGCTACTACTGC
PIR1-RT-R	ACCAGTTGCAATTGCTTGAC
RIP1-RT-F	TGTACCCACTTGGGTTGTGT
RIP1-RT-R	ATGGAGCTGGACCCTTTCTA
RPL13-RT-F	TGCTAACGTCGCCAGATTAC
RPL13-RT-R	GGTTGTTCAACTGGGAAGGT

File S1

Fold changes and p-values of each transcript in every condition. Each average fold change was calculated as the ratio of the mean normalized RPKM value in the treated condition over the mean normalized RPKM value in the time-matched untreated condition. P-values represent significance of differential expression according to a PLGEM analysis (Pavelka et al. 2004).

File S1 is available for download at http://www.g3journal.org/lookup/suppl/doi:10.1534/g3.114.015941/-/DC1

File S2

GO enrichment in genes regulated in the different conditions. Enrichment of GO Biological Process terms was performed on all lists of genes significantly regulated at any time point (T1, T2, T3 or T4) in response to lactic, acetic, propionic or butyric acid, following removal of genes significantly regulated by HCl. Values correspond to multiple-testing corrected p-values obtained from CGD (Inglis et al. 2012). Only p-value ≤ 0.1 are reported. Positive values refer to enrichment within the up-regulated genes, while negative values indicate enrichment among the down-regulated genes.

File S2 is available for download at http://www.g3journal.org/lookup/suppl/doi:10.1534/g3.114.015941/-/DC1

File S3

Additional Materials and Methods

Quantitative real-time PCR analysis. RNA extraction for validation of transcript expression was performed using the Qiagen RNeasy kit (Qiagen) and cDNA was then synthesized using the Superscript III kit (Invitrogen) in 20 µl reaction volumes starting with ~100 ng of total RNA per sample as per manufacturers' protocol. qPCR reactions were then set up in 384-well plates and cycled using an ABI 7900HT (Applied Biosystems) in 10 µl reaction volumes as described previously (Pavelka et al. 2010) using primers listed in Tables S3.

References

Inglis, D.O., M.B. Arnaud, J. Binkley, P. Shah, M.S. Skrzypek *et al.*, 2012 The Candida genome database incorporates multiple Candida species: multispecies search and analysis tools with curated gene and protein information for Candida albicans and Candida glabrata. *Nucleic Acids Res.* 40 (Database issue):D667-674.

Pavelka, N., M. Pelizzola, C. Vizzardelli, M. Capozzoli, A. Splendiani *et al.*, 2004 A power law global error model for the identification of differentially expressed genes in microarray data. *BMC Bioinformatics* 5:203.

Pavelka, N., G. Rancati, J. Zhu, W.D. Bradford, A. Saraf *et al.*, 2010 Aneuploidy confers quantitative proteome changes and phenotypic variation in budding yeast. *Nature* 468 (7321):321-325.
