# Supplementary material for: The Transcriptional Stress Response of Candida albicans to Weak Organic Acids
Source: G3 (Bethesda). 2015 Jan 29;5(4):497–505. doi: 10.1534/g3.114.015941 (PMC4390566; doi:10.1534/g3.114.015941)
Supplement: Supporting Information [file supp_g3.114.015941_FigureS1.pdf]

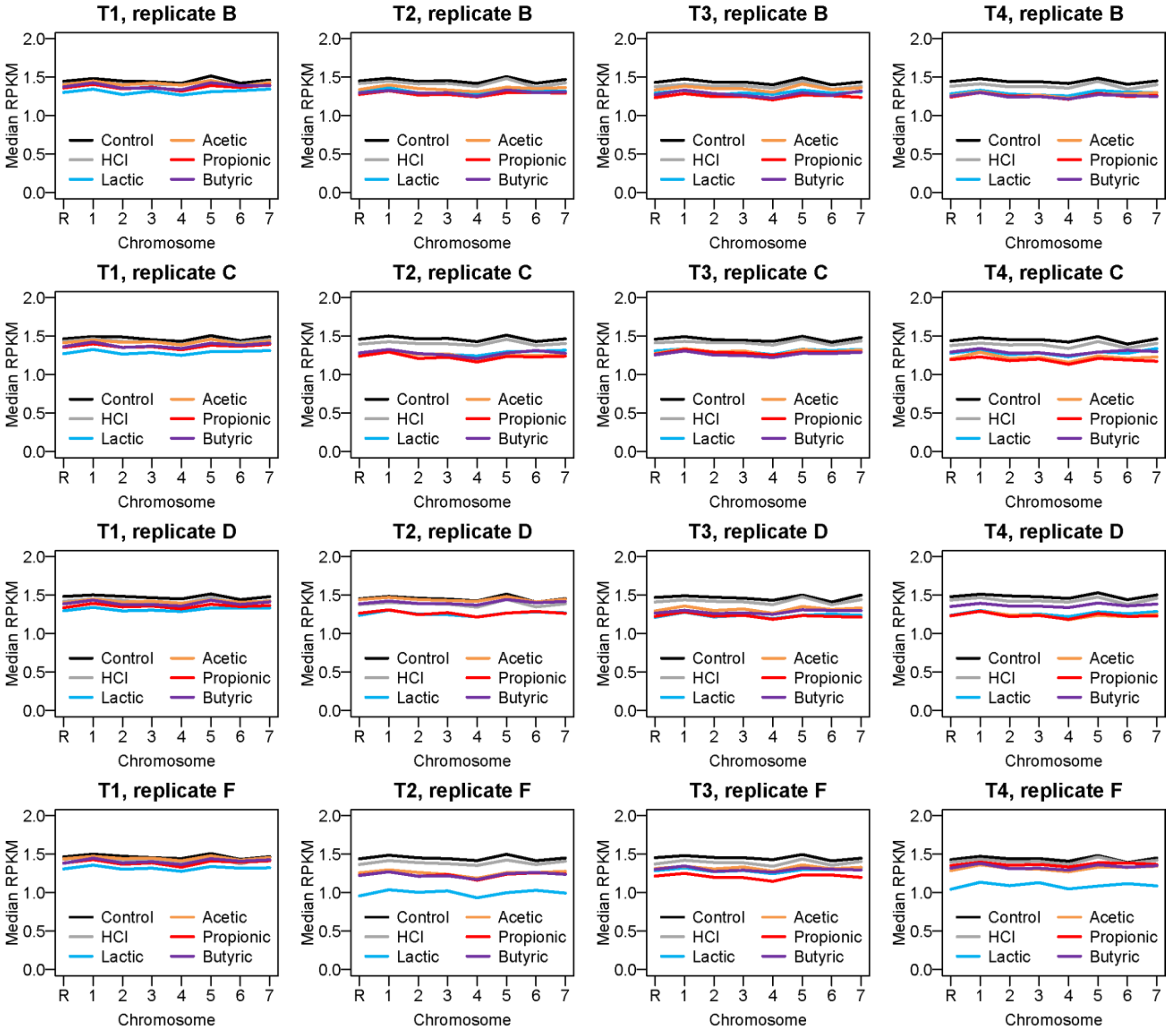

Figure S1 No evidence for aneuploidy in any sequenced sample. The median expression value (log10-transformed RPKM) of each of the eight chromosomes in the *C. albicans* genome is plotted for each of the 96 sequenced transcriptome samples. Samples are grouped by time point (column) and replicate (row). None of the samples displayed observable deviations from uniformity between the chromosomes, which would have otherwise indicated a possible presence of aneuploidy.
