# Supplementary material for: The Transcriptional Stress Response of Candida albicans to Weak Organic Acids
Source: G3 (Bethesda). 2015 Jan 29;5(4):497–505. doi: 10.1534/g3.114.015941 (PMC4390566; doi:10.1534/g3.114.015941)
Supplement: Supporting Information [file supp_g3.114.015941_FigureS2.pdf]

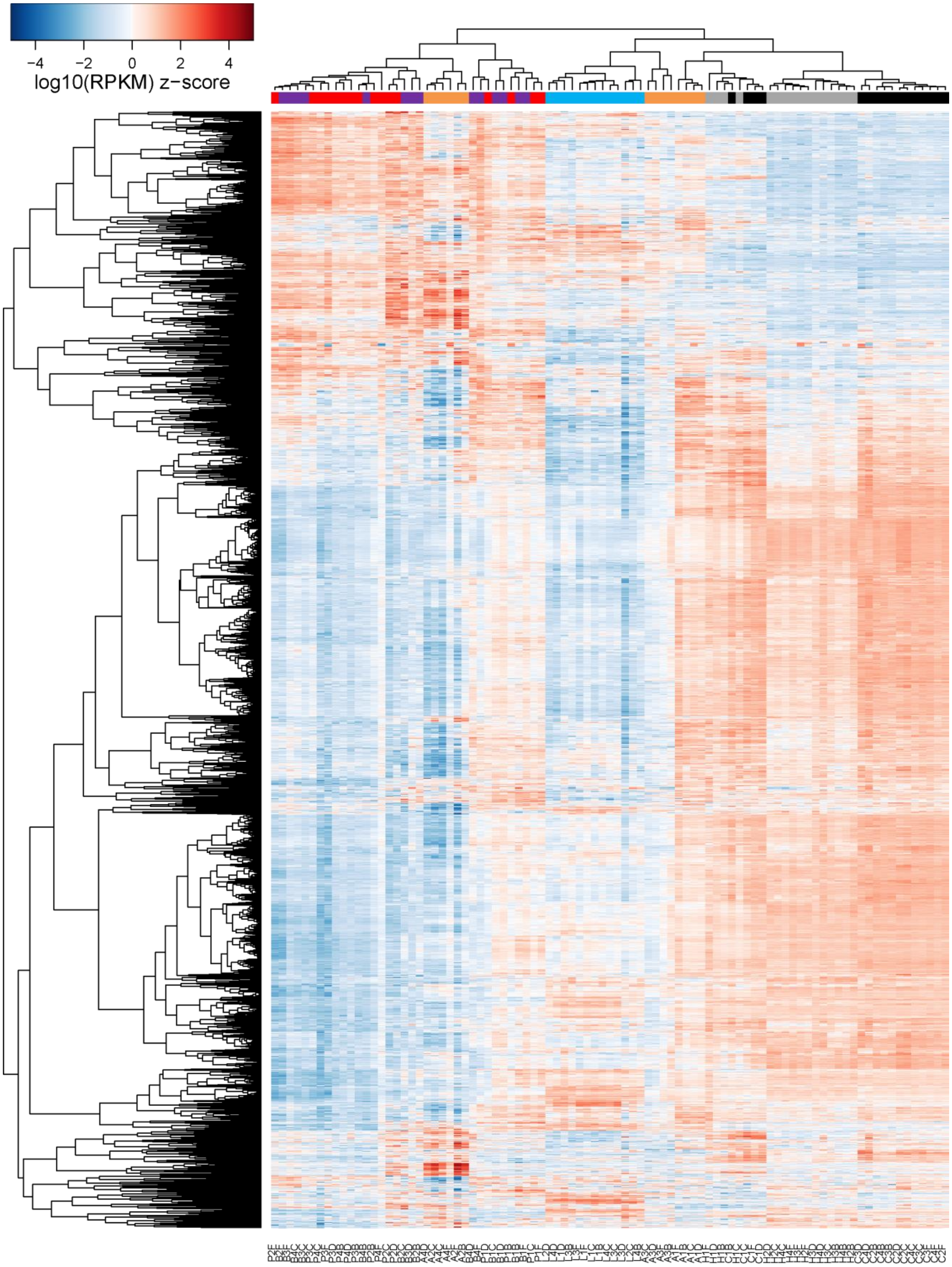

Figure S2 Global transcriptional response of *C. albicans* to WOAs. The figure displays a two-way hierarchical clustering of 6,453 transcripts (rows) and 89 samples (columns). Sample names are indicated at the bottom and follow the same nomenclature described in the legend of Figure 2. Samples labeled as L2F, L4F, L2B, A4B, B2D, A2D and B3B were discarded as outliers from this and all other analyses. Log10-transformed RPKM expression values were converted to z-scores, with red indicating expression levels above and blue symbolizing expression levels below the mean expression level of each gene across the samples. Distance metric = 1 − Pearson correlation.
