# Supplementary material for: The Transcriptional Stress Response of Candida albicans to Weak Organic Acids
Source: G3 (Bethesda). 2015 Jan 29;5(4):497–505. doi: 10.1534/g3.114.015941 (PMC4390566; doi:10.1534/g3.114.015941)
Supplement: Supporting Information [file supp_g3.114.015941_FigureS3.pdf]

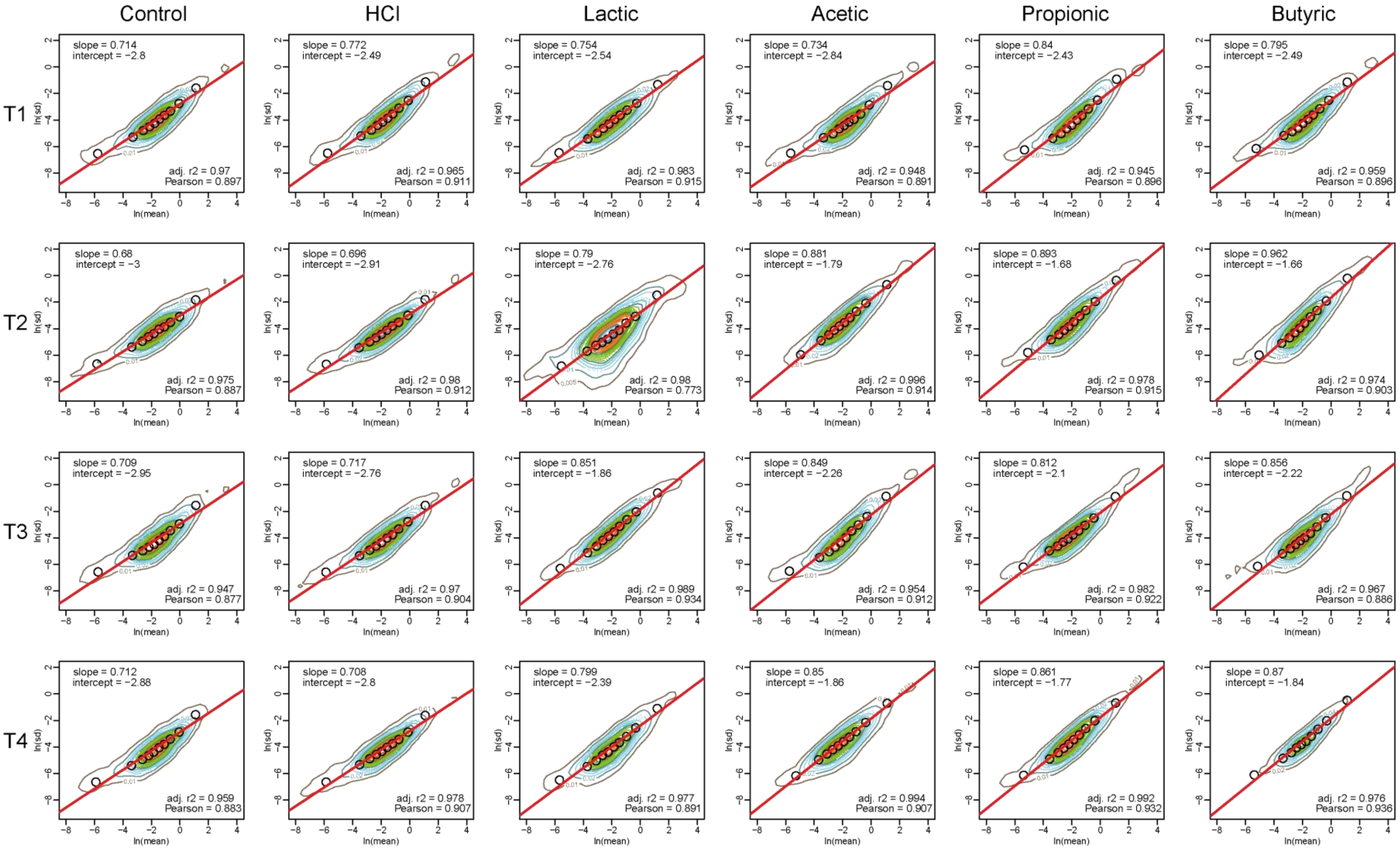

Figure S3 PLGEM goodness-of-fit. A PLGEM was fitted to each set of biological replicates corresponding to an individual treatment (column) and time point (row). For each set of replicates, the logarithm of the standard deviation of the normalized RPKM values [ln(sd)] was plotted against the logarithm of the mean normalized RPKM value [ln(mean)] calculated for each of 6,453 transcripts. Diagnostic plots of the goodness-of-fit include a contour plot of all analyzed transcripts, ten modeling points (black circles) and a linear regression representing the PLGEM (red line). Inset numbers in each plot represent the slope, the intercept and the adjusted r^2 of the linear regression based on the modeling points, as well as the Pearson correlation of all ln(sd) vs. ln(mean) values. Details of the PLGEM algorithm and a definition and interpretation of all above parameters have been previously described (Pavelka et al. 2004).
