# Supplementary material for: The Transcriptional Stress Response of Candida albicans to Weak Organic Acids
Source: G3 (Bethesda). 2015 Jan 29;5(4):497–505. doi: 10.1534/g3.114.015941 (PMC4390566; doi:10.1534/g3.114.015941)
Supplement: Supporting Information [file supp_g3.114.015941_FigureS4.pdf]

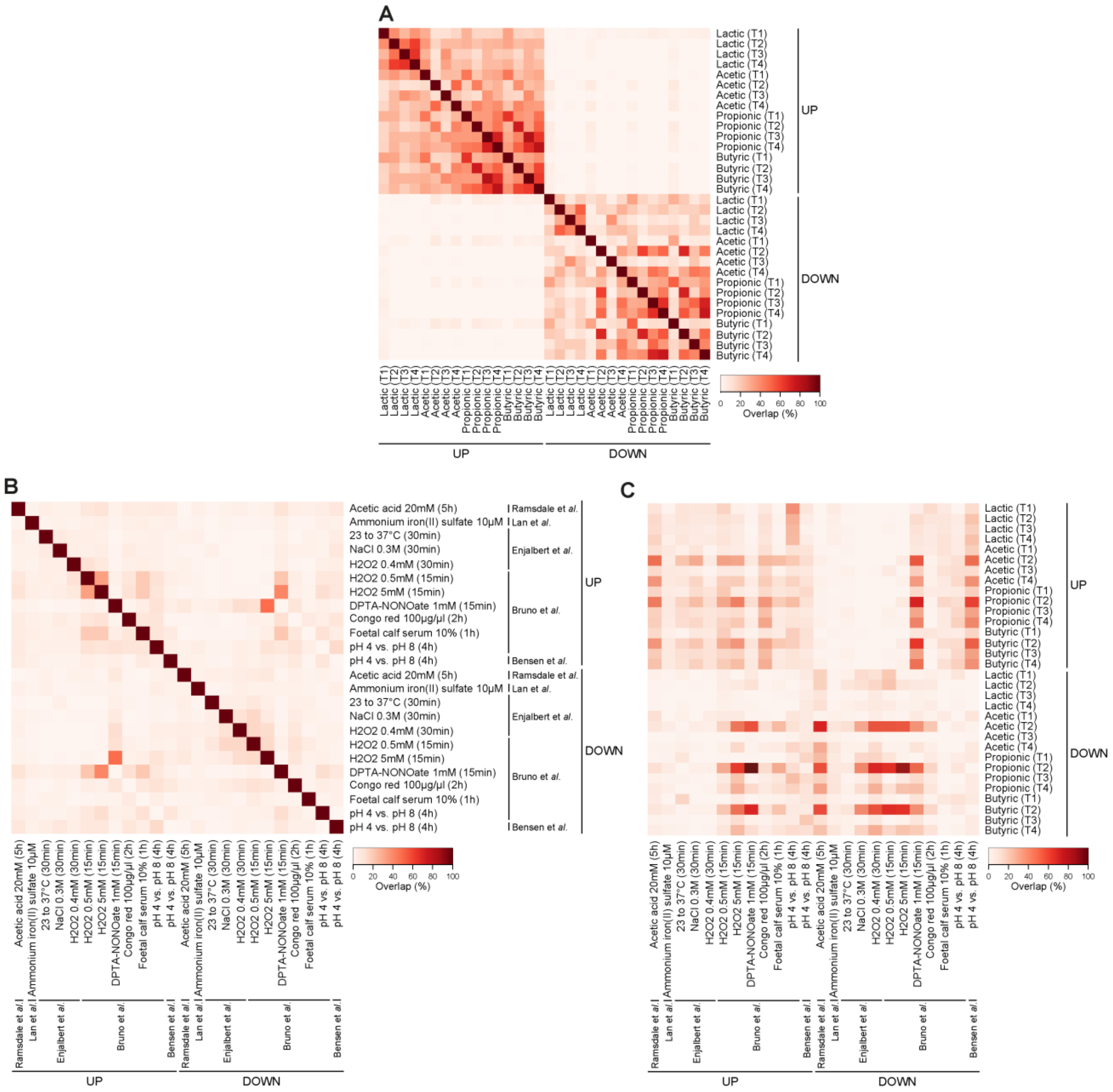

Figure S4 Comparison of *C. albicans* transcriptional response to WOA and other transcriptional stress responses. (A-C) Pair-wise overlap analysis between individual lists of genes up- or down-regulated under the indicated conditions. Each heat map is color-coded based on the percentage of overlap between the indicated pair of gene lists according to the color key next to each heat map. Samples are identical to the ones presented on Figure 3.
