# Supplementary material for: The Transcriptional Stress Response of Candida albicans to Weak Organic Acids
Source: G3 (Bethesda). 2015 Jan 29;5(4):497–505. doi: 10.1534/g3.114.015941 (PMC4390566; doi:10.1534/g3.114.015941)
Supplement: Supporting Information [file supp_g3.114.015941_FigureS5.pdf]

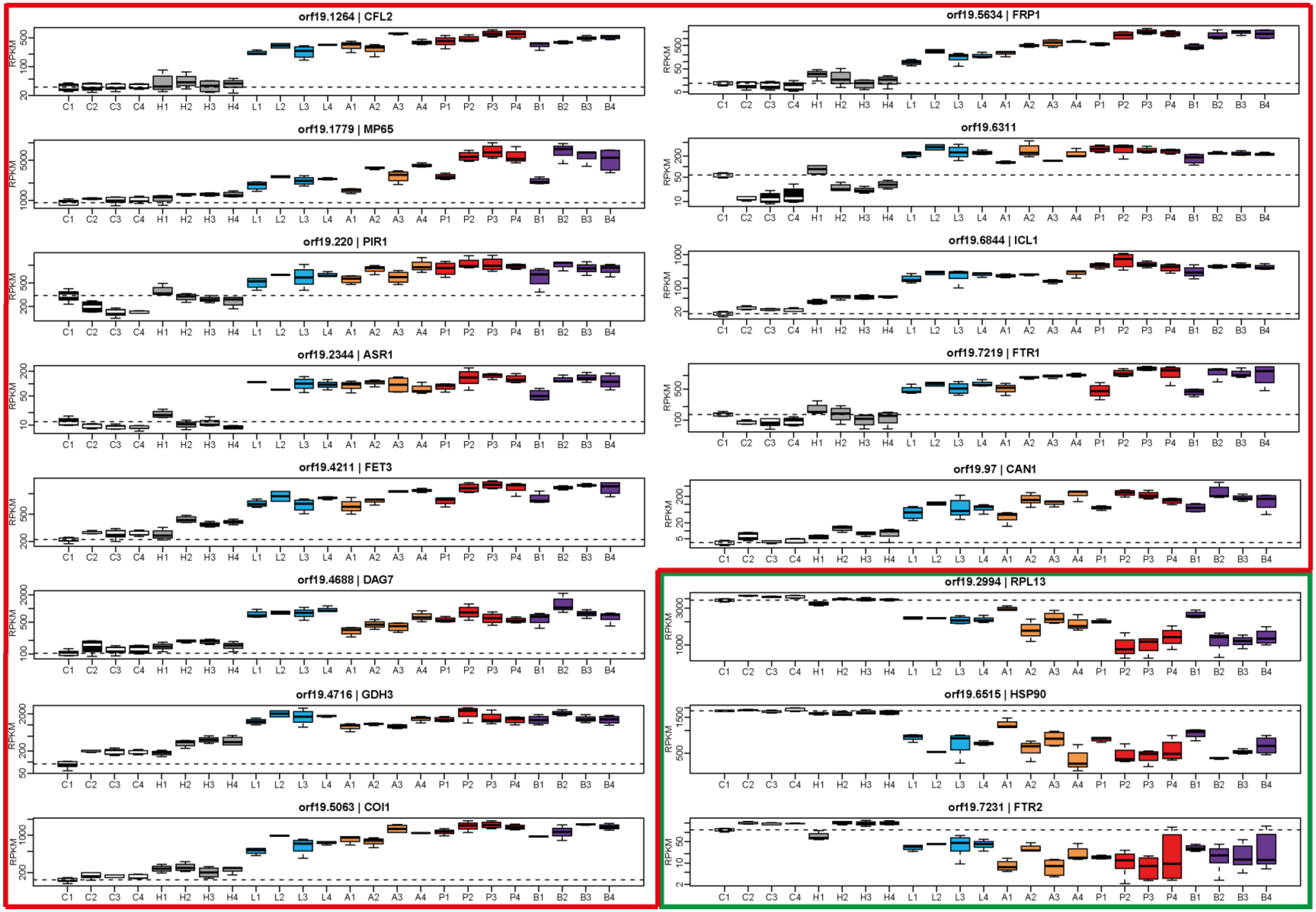

Figure S5 Expression levels of the 16 core genes commonly regulated by all WOAs at all times. Genes included in the red frame were up-regulated in presence of all WOAs throughout the time course, while the genes framed in green were consistently down-regulated. Data are represented as boxplots to show the distribution across the biological replicates.
