# Supplementary material for: The Transcriptional Stress Response of Candida albicans to Weak Organic Acids
Source: G3 (Bethesda). 2015 Jan 29;5(4):497–505. doi: 10.1534/g3.114.015941 (PMC4390566; doi:10.1534/g3.114.015941)
Supplement: Supporting Information [file supp_g3.114.015941_FigureS6.pdf]

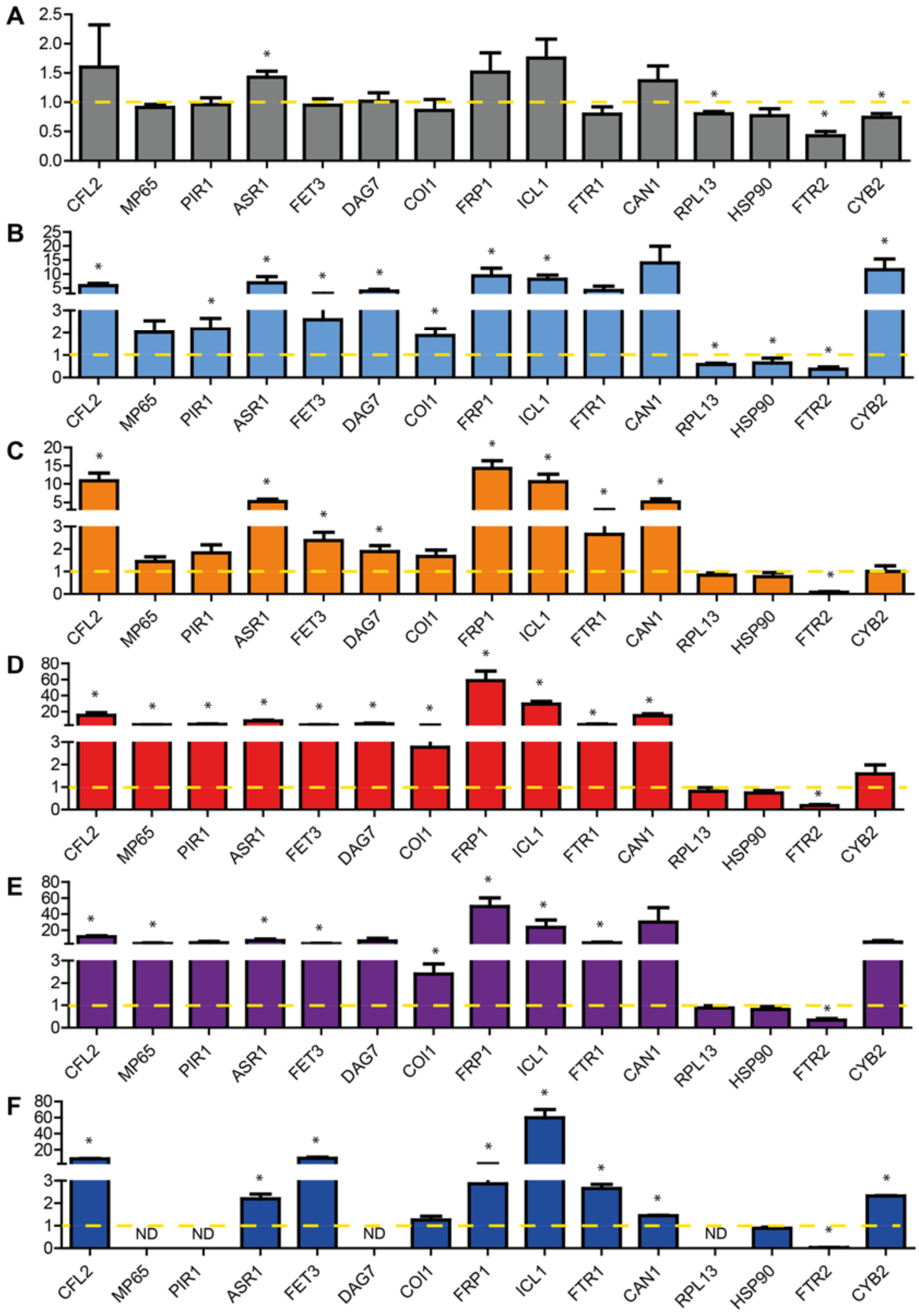

Figure S6 Quantitative RT-PCR validates gene regulation observed by RNA-sequencing. Relative expression levels of the indicated genes were normalized to the *RIP1* endogenous control to confirm significant regulation during exposure to HCl (A), lactic (B), acetic (C), propionic (D) and butyric acid (E) at pH 5.5 compared to untreated control cultures (MRS). (A-E) qPCR performed on same cDNA used for RNA-seq. (F) Similar experiment under exposure to lactic acid (62 mM) at pH 4.5 compared to the control (MRS). Statistical analysis was performed in Microsoft Excel, using one-sample t-test assuming equal variances. $n \geq 3$; * $p < 0.05$
