# Supplementary material for: The Transcriptional Stress Response of Candida albicans to Weak Organic Acids
Source: G3 (Bethesda). 2015 Jan 29;5(4):497–505. doi: 10.1534/g3.114.015941 (PMC4390566; doi:10.1534/g3.114.015941)
Supplement: Supporting Information [file supp_g3.114.015941_TableS1.pdf]

Table S1 Genes significantly regulated in response to each WOA at all time points. Genes differentially expressed in response to lactic, acetic, propionic or butyric acid at T1, T2, T3 and T4. These lists were used to draw the Venn diagram in Figure 3D. Transcript identifiers refer to the *C. albicans* Assembly 21 genome annotation.

Weak organic acid	Genes significantly regulated in the same direction at all time-points
Lactic	orf19.1075.1, orf19.125, orf19.1264, orf19.1358, orf19.1480, orf19.1779, orf19.1857, orf19.2021, orf19.2066.1, orf19.220, orf19.2344, orf19.251, orf19.2525, orf19.2765, orf19.3160, orf19.3171, orf19.3340, orf19.3548.1, orf19.3954.1, orf19.4211, orf19.4216, orf19.4506, orf19.4551, orf19.4688, orf19.4716, orf19.5000, orf19.5005, orf19.5032, orf19.5063, orf19.5213.1, orf19.5517, orf19.5610, orf19.5634, orf19.5653, orf19.5753, orf19.5805, orf19.6165, orf19.6311, orf19.6724, orf19.6757, orf19.6844, orf19.6854, orf19.691, orf19.7219, orf19.7279.1, orf19.7398.1, orf19.7417, orf19.7469, orf19.789, orf19.97, ITS2, orf19.1030, orf19.1389, orf19.1697, orf19.200, orf19.2111.2, orf19.2767, orf19.2994, orf19.3572.3, orf19.3810, orf19.4054, orf19.4336, orf19.4492, orf19.4623, orf19.4660, orf19.4909.1, orf19.493, orf19.5225.2,orf19.5351, orf19.5904, orf19.5943.1, orf19.5964.2, orf19.6220.4, orf19.6414.3, orf19.6515, orf19.7018, orf19.7046, orf19.7231, orf19.7477 ,orf19.827.1, orf19.962
Acetic	orf19.1263, orf19.1264, orf19.1395, orf19.1779, orf19.2062, orf19.220, orf19.2344, orf19.2762, orf19.3160, orf19.3461, orf19.3548.1, orf19.4211, orf19.4216, orf19.4505, orf19.4688, orf19.4716, orf19.4802, orf19.5063, orf19.5634, orf19.5952, orf19.6073, orf19.6116, orf19.6311, orf19.6844, orf19.7021, orf19.7111.1, orf19.7219, orf19.734, orf19.97, orf19.1065, orf19.1517, orf19.238, orf19.2767, orf19.2994, orf19.3433, orf19.386, orf19.4674.1, orf19.6515, orf19.6948, orf19.7046, orf19.7231
Propionic	orf19.1030, orf19.1065, orf19.1154, orf19.1470, orf19.1517, orf19.1601, orf19.1697, orf19.1700, orf19.1770, orf19.1853, orf19.1996, orf19.2067, orf19.2091, orf19.2111.2, orf19.2179.2, orf19.2232, orf19.2310.1, orf19.2329.1, orf19.236, orf19.238, orf19.2422, orf19.2451, orf19.2478.1, orf19.269, orf19.2709, orf19.2767, orf19.2859, orf19.2864.1, orf19.2871, orf19.2935, orf19.2948, orf19.2994, orf19.3002, orf19.3034, orf19.3087, orf19.3138, orf19.3175, orf19.3325.3, orf19.336, orf19.3396, orf19.3415.1, orf19.3426, orf19.3433, orf19.3572.3, orf19.3788.1, orf19.3789, orf19.3810, orf19.3812, orf19.385, orf19.386, orf19.3870, orf19.3962, orf19.4026, orf19.4040, orf19.4060, orf19.4093, orf19.4336, orf19.4375.1, orf19.4447, orf19.4468, orf19.4490, orf19.4492, orf19.4622, orf19.4623, orf19.4660, orf19.4674.1, orf19.4758, orf19.4909.1, orf19.493, orf19.5077, orf19.5126, orf19.5225.2, orf19.526, orf19.5341, orf19.5351, orf19.5466, orf19.5750, orf19.5838, orf19.5858, orf19.5904, orf19.5943.1, orf19.5949, orf19.5964.2, orf19.6002, orf19.6085, orf19.6090, orf19.6220.4, orf19.6253, orf19.6264.4, orf19.6265, orf19.6286.2, orf19.6375, orf19.637, orf19.6515, orf19.6541, orf19.6632, orf19.6702, orf19.6749, orf19.6785, orf19.687.1, orf19.6948, orf19.7018, orf19.7046, orf19.7048.1, orf19.7050, orf19.7161, orf19.7217, orf19.7231, orf19.7236, orf19.7397.1, orf19.7424, orf19.7477, orf19.7654, orf19.827.1, orf19.838.1, orf19.1075.1, orf19.1149, orf19.1227, orf19.1237, orf19.125, orf19.1263, orf19.1264, orf19.1339, orf19.1395, orf19.1690, orf19.1779, orf19.1891, orf19.1974, orf19.2062, orf19.2125, orf19.2158, orf19.2179, orf19.220, orf19.2344, orf19.23, orf19.2608, orf19.2686, orf19.2762, orf19.2990, orf19.3053, orf19.3104, orf19.3234, orf19.3340, orf19.3378, orf19.3461, orf19.3507, orf19.3538, orf19.3612, orf19.36.1, orf19.3732, orf19.4082, orf19.4211, orf19.4505, orf19.4565, orf19.4630, orf19.4688, orf19.4716, orf19.4802, orf19.4833, orf19.4886, orf19.4905, orf19.5024, orf19.5063, orf19.5112, orf19.5379, orf19.5517, orf19.5610, orf19.5634, orf19.5653, orf19.5683, orf19.5686, orf19.5730, orf19.5777, orf19.5791, orf19.5801, orf19.5805, orf19.5952, orf19.6073, orf19.6116, orf19.6311, orf19.6318, orf19.638, orf19.6840, orf19.6844, orf19.6854, orf19.6877, orf19.6951, orf19.7021, orf19.7111.1, orf19.7196, orf19.7219, orf19.7279.1, orf19.7306, orf19.734, orf19.7398.1, orf19.7417, orf19.7469, orf19.7531, orf19.7611, orf19.7676, orf19.767, orf19.822, orf19.97
Butyric	orf19.1030, orf19.1065, orf19.1517, orf19.1601, orf19.1697, orf19.2111.2, orf19.238, orf19.2451, orf19.2864.1, orf19.2994, orf19.3002, orf19.3175, orf19.3572.3, orf19.3810, orf19.385, orf19.386, orf19.4040, orf19.4336, orf19.4447, orf19.4660, orf19.4674.1, orf19.4909.1, orf19.493, orf19.5225.2, orf19.5466, orf19.5750, orf19.5858, orf19.5904, orf19.5943.1, orf19.5964.2, orf19.6090, orf19.6220.4, orf19.6515, orf19.6541, orf19.6632, orf19.687.1, orf19.6948, orf19.7018, orf19.7231, orf19.827.1, orf19.838.1, orf19.1048, orf19.1075.1, orf19.1149, orf19.1227, orf19.1237, orf19.125, orf19.1263, orf19.1264, orf19.1339, orf19.1395, orf19.1690, orf19.1779, orf19.1891, orf19.1974, orf19.2062, orf19.2158, orf19.2160, orf19.220, orf19.2344, orf19.2762, orf19.3104, orf19.3234, orf19.3340, orf19.3378, orf19.3461, orf19.3507, orf19.3612, orf19.36.1, orf19.4211, orf19.4505, orf19.4565, orf19.4609, orf19.4688, orf19.4716, orf19.4773, orf19.4802, orf19.4833, orf19.4886, orf19.4905, orf19.5063, orf19.5112, orf19.5379, orf19.5517, orf19.5610, orf19.5634, orf19.5653, orf19.5683, orf19.5686, orf19.5730, orf19.5791, orf19.5801, orf19.5805, orf19.5952, orf19.6073, orf19.6116, orf19.6311, orf19.6318, orf19.638, orf19.6844, orf19.6854, orf19.6877, orf19.6951, orf19.7111.1, orf19.7112, orf19.7196, orf19.7219, orf19.7279.1, orf19.7306, orf19.734, orf19.7398.1, orf19.7417, orf19.7469, orf19.7531, orf19.7611, orf19.822, orf19.97
