# Supplementary material for: The Transcriptional Stress Response of Candida albicans to Weak Organic Acids
Source: G3 (Bethesda). 2015 Jan 29;5(4):497–505. doi: 10.1534/g3.114.015941 (PMC4390566; doi:10.1534/g3.114.015941)
Supplement: Supporting Information [file supp_g3.114.015941_TableS2.pdf]

Table S2 Comparison of the 16 WOA core response genes to previously published *C. albicans* transcriptional stress responses. The presence of each of the 16 core response genes to WOAs (13 up-regulated and 3 down-regulated genes) in previously published gene lists was evaluated. A bullet symbolizes that the indicated WOA core response transcript was found to be at least two-fold up-regulated (A) or down-regulated (B) in the previously published *C. albicans* transcriptional stress response indicated in the corresponding column header. Genes consistently up-regulated (A) or consistently down-regulated (B) in both gene lists are highlighted in pink (A) or light blue (B), respectively.

(A) Genes up-regulated in previously published *C. albicans* stress responses.

**WOA core response**			**Acetic acid 20mM (5h)**	**Ammonium iron(II) sulfate 10µM**	**23 to 37 °C (30min)**	**NaCl 0.3M (30min)**	**H_2O_2 0.4mM (30min)**	**H_2O_2 0.5mM (15min)**	**H_2O_2 5mM (15min)**	**DPTA-NONOate 1mM (15min)**	**Congo red 100µg/µl (2h)**	**Foetal calf serum 10% (1h)**	**pH 4 vs. pH 8 (4h) [Bruno et al.]**	**pH 4 vs. pH 8 (4h) [Bensen et al.]**
UP	orf19.1264	CFL2		•				•		•				•
	orf19.1779	MP65												•
	orf19.220	PIR1	•											
	orf19.2344	ASR1			•	•		•	•		•	•	•	
	orf19.4211	FET3								•				
	orf19.4688	DAG7				•				•			•	
	orf19.4716	GDH3						•						
	orf19.5063	COI1		•							•			
	orf19.5634	FRP1		•				•			•	•		
	orf19.6311	orf19.6311	•		•			•	•	•				
	orf19.6844	ICL1	•					•	•				•	
	orf19.7219	FTR1						•		•	•			
	orf19.97	CAN1	•				•	•			•		•	
DOWN	orf19.2994	RPL13												•
	orf19.6515	HSP90												
	orf19.7231	FTR2								•				•

(B) Genes down-regulated in previously published *C. albicans* stress responses.

WOA core response			Acetic acid 20mM (5h)	Ammonium iron(II) sulfate 10µM	23 to 37 °C (30min)	NaCl 0.3M (30min)	H_2O_2 0.4mM (30min)	H_2O_2 0.5mM (15min)	H_2O_2 5mM (15min)	DPTA-NONOate 1mM (15min)	Congo red 100µg/µl (2h)	Foetal calf serum 10% (1h)	pH 4 vs. pH 8 (4h) [Bruno et al.]	pH 4 vs. pH 8 (4h) [Bensen et al.]
UP	orf19.1264	CFL2											•	
	orf19.1779	MP65							•					
	orf19.220	PIR1		•				•				•		
	orf19.2344	ASR1												•
	orf19.4211	FET3			•	•			•		•	•	•	
	orf19.4688	DAG7			•			•	•		•			
	orf19.4716	GDH3				•			•	•		•	•	
	orf19.5063	COI1												
	orf19.5634	FRP1							•	•			•	
	orf19.6311	orf19.6311												
	orf19.6844	ICL1								•				
	orf19.7219	FTR1							•					
	orf19.97	CAN1												
DOWN	orf19.2994	RPL13	•											
	orf19.6515	HSP90												
	orf19.7231	FTR2		•	•		•	•	•		•			
