# Supplementary material for: The Transcriptional Stress Response of Candida albicans to Weak Organic Acids
Source: G3 (Bethesda). 2015 Jan 29;5(4):497–505. doi: 10.1534/g3.114.015941 (PMC4390566; doi:10.1534/g3.114.015941)
Supplement: Supporting Information [file supp_g3.114.015941_TableS3.pdf]

Table S3 Quantitative RT-PCR primers used in this study.

Primer name	Sequence
ASR1-RT-F	ATGGCGATTCCAGTTATGGT
ASR1-RT-R	GCATCAAGATGGTCAACACC
CAN1-RT-F	CAAGCTGGTGTTGCTCCTAA
CAN1-RT-R	TGACTGGAACAGGCCAAGTA
CFL2-RT-F	TATCGAAGGACGTCCAAACA
CFL2-RT-R	AGGATGTCCACACGTAACGA
COI1-RT-F	TGGATGTTATGAAACAACAAGATG
COI1-RT-R	ATTTAGCACAAGCACCACCA
CYB2-RT-F	TACGGTTGATGCTCCACAAT
CYB2-RT-R	AAATGGCTCTTGCTGAACCT
DAG7-RT-F	CTCCATCAACAACTTCAGCAA
DAG7-RT-R	CAACAATGAAATCGGAATCG
FET3-RT-F	GCCATATTGAATGGCATTTG
FET3-RT-R	GGCATTACCTTCCCAGGATA
FRP1-RT-F	TTTGGTGCAACACCGTATTT
FRP1-RT-R	AACCCTGTCGATAACCAACC
FTR1-RT-F	ACTGGTGGTGATGCTTCTGA
FTR1-RT-R	CCGTTATCGGTTTCTGGATT
FTR2-RT-F	AATTTGTGGTCTTGCAGTGG
FTR2-RT-R	CTCTGGAGAACAAACCAGCA
HSP90-RT-F	TACGAACCATTGACCAAAGC
HSP90-RT-R	CAGACCAACCAAATTGACCA
ICL1-RT-F	AGAAATTGGCCAAGGAATTG
ICL1-RT-R	GACGGCACATTGAGTACCAC
MP65-RT-F	AACTCTGGTGCTTGGGTCTT
MP65-RT-R	TAGATGGCCAACCAGTTTCA
PIR1-RT-F	GCTGCTGCTGCTACTACTGC
PIR1-RT-R	ACCAGTTGCAATTGCTTGAC
RIP1-RT-F	TGTACCCACTTGGGTTGTGT
RIP1-RT-R	ATGGAGCTGGACCCTTTCTA
RPL13-RT-F	TGCTAACGTCGCCAGATTAC
RPL13-RT-R	GGTTGTTCAACTGGGAAGGT
