# Supplementary material for: The Transcriptional Stress Response of Candida albicans to Weak Organic Acids
Source: G3 (Bethesda). 2015 Jan 29;5(4):497–505. doi: 10.1534/g3.114.015941 (PMC4390566; doi:10.1534/g3.114.015941)
Supplement: Supporting Information [file supp_g3.114.015941_FileS3.pdf]

File S3

Additional Materials and Methods

Quantitative real-time PCR analysis. RNA extraction for validation of transcript expression was performed using the Qiagen RNeasy kit (Qiagen) and cDNA was then synthesized using the Superscript III kit (Invitrogen) in 20 µl reaction volumes starting with ~100 ng of total RNA per sample as per manufacturers' protocol. qPCR reactions were then set up in 384-well plates and cycled using an ABI 7900HT (Applied Biosystems) in 10 µl reaction volumes as described previously (Pavelka et al. 2010) using primers listed in Tables S3.
